# Water channel protein AQP1 in cytoplasm is a critical factor in breast cancer local invasion

**DOI:** 10.1186/s13046-023-02616-1

**Published:** 2023-02-20

**Authors:** Zhifang Guo, Huikun Zhang, Xiaoli Liu, Yawen Zhao, Yongzi Chen, Jiaqi Jin, Caixia Guo, Ming Zhang, Feng Gu, Yongjie Ma

**Affiliations:** 1 Department of Tumor Cell Biology, Tianjin Medical University Cancer Institute and Hospital, National Clinical Research Center for Cancer, Huanhu West Road, Hexi District, Tianjin, 300060 People’s Republic of China; 2 Tianjin’s Clinical Research Center for Cancer, Tianjin Medical University Cancer Institute and Hospital, Tianjin, China; 3 Key Laboratory of Cancer Prevention and Therapy, Tianjin, China; 4 Key Laboratory of Breast Cancer Prevention and Therapy, Tianjin Medical University, Ministry of Education, Tianjin, China; 5 CAS Key Laboratory of Genomics and Precision Medicine, Beijing Institute of Genomics, University of Chinese Academy of Sciences, Chinese Academy of Sciences, China National Center for Bioinformation, Beijing, 100101 China; 6 Department of Epidemiology and Biostatistics, University of Georgia, Athens, GA USA; 7 Department of Breast Cancer Pathology and Research Laboratory, Tianjin Medical University Cancer Institute and Hospital, Tianjin, China

**Keywords:** Breast cancer, AQP1, Metastasis

## Abstract

**Background:**

Metastasis of breast cancer grows from the local invasion to the distant colonization. Blocking the local invasion step would be promising for breast cancer treatment. Our present study demonstrated AQP1 was a crucial target in breast cancer local invasion.

**Methods:**

Mass spectrometry combined with bioinformatics analysis was used to identify AQP1 associated proteins ANXA2 and Rab1b. Co-immunoprecipitation, immunofluorescence assays and cell functional experiments were carried out to define the relationship among AQP1, ANXA2 and Rab1b and their re-localization in breast cancer cells. The Cox proportional hazards regression model was performed toward the identification of relevant prognostic factors. Survival curves were plotted by the Kaplan–Meier method and compared by the log-rank test.

**Results:**

Here, we show that the cytoplasmic water channel protein AQP1, a crucial target in breast cancer local invasion, recruited ANXA2 from the cellular membrane to the Golgi apparatus, promoted Golgi apparatus extension, and induced breast cancer cell migration and invasion. In addition, cytoplasmic AQP1 recruited cytosolic free Rab1b to the Golgi apparatus to form a ternary complex containing AQP1, ANXA2, and Rab1b, which induced cellular secretion of the pro-metastatic proteins ICAM1 and CTSS. Cellular secretion of ICAM1 and CTSS led to the migration and invasion of breast cancer cells. Both in vivo assay and clinical analysis data confirmed above results.

**Conclusions:**

Our findings suggested a novel mechanism for AQP1-induced breast cancer local invasion. Therefore, targeting AQP1 offers promises in breast cancer treatment.

**Supplementary Information:**

The online version contains supplementary material available at 10.1186/s13046-023-02616-1.

## Background

The most common cause of breast cancer-related death is metastasis [1]. Currently, no targeted therapies for breast cancer metastasis are available despite extensive research. Metastasis of breast cancer spreads from a local site to distant colonization sites through the surrounding extracellular matrix, stromal cell layers, and blood vessels [2]. Local invasion refers to the entry of tumor cells into the surrounding tumor-associated stroma and then into the adjacent normal tissue parenchyma. A critical step in invading the stroma is that cancer cells must first breach the basement membrane (BM), acting as a barrier between the epithelial cells and the surrounding stroma. BMs are sheet-like cell-adherent extracellular matrices (ECMs) that can be released by cancer-secreted proteases. Given the importance of the local invasion in metastasis, blocking this step would be promising for breast cancer treatment.

The association between the expression of AQP1 and cancer progression have been reported. Hoque et al. found that AQP1 was overexpressed in lung cancer and promoted cell proliferation and anchorage-independent growth [3]. In osteosarcoma and hepatocellular carcinoma, inhibition of AQP1 hampered tumor cell migration and invasion [4]. Inhibition of AQP1 reduced tumor growth in tumor-producing MMTV-PyVT mice or a mouse model of melanoma [5]. Our previous reports indicated that the water channel protein AQP1 was localized dominantly in the cytoplasm of breast cancer cells, and expression of AQP1 was positively correlated with advanced pathological features of invasive ductal carcinoma (IDC). Cytoplasmic expression of AQP1 in lymph node metastases was higher than their paired primary tumors and cytoplasmic AQP1 was an independent prognostic factor, high expression of AQP1 indicated a poor prognosis [6]. There were several reports which were consistent with our results. Shi Z, et al. showed that the expression of AQP1 in tumor cells was increased in comparison to adjacent normal tissue in breast cancer patients [7]. Otterbach’s group reported AQP1 expression was positive associated with poor survival, where high expression of AQP1 was positive associated with high tumor grade in breast cancer [8]. However, it remains unclear how cytoplasmic AQP1 effects breast cancer malignant progression. The present study focused on the molecular mechanisms of cytoplasmic AQP1 in promoting breast cancer development.

Here we identified the water channel protein AQP1 as a crucial target in breast cancer cell invasion. AQP1 can also reside in the Golgi apparatus, where it can induce cell secretion of ICAM1 and CTSS, leading to the local invasion of breast cancer. We found that AQP1 recruited ANXA2 to the Golgi apparatus, promoted Golgi apparatus extension, and induced secretion of ICAM1 and CTSS, which induced breast cancer cell invasion. Meanwhile, cytoplasmic AQP1 also recruited cytosolic free Rab1b to the Golgi apparatus and bound with Rab1b within the Golgi apparatus, which promoted cell secretion of ICAM1 and CTSS. During this process, Rab1b interacted through its C-terminal domain with ANXA2. Finally, AQP1, ANXA2, and Rab1b could form a ternary complex in the Golgi apparatus and induce cell secretion of prometastatic proteins ICAM1 and CTSS, which leads to the local invasion.

In conclusion, this study demonstrates that AQP1 is critical for the local invasion in breast cancer metastasis. Therefore, targeting AQP1 offers promises in breast cancer treatment.

## Materials and methods

### Bioinformatics analysis

RNA-seq gene expression data for 817 breast cancer patients showed in Fig. 6a, Supplementary Figs. 4a-e and 5f were downloaded from cBioportal (http://www.cbioportal.org/) [9]. The samples were ranked in ascending order by the values of expression of AQP1 gene, the expression levels of AQP1 gene were defined based on ranking (the top 200 of samples have high expression, and the bottom 200 have low expression). Besides, in order to analyze the ANXA2-related genes, the values of expression of ANXA2 gene were also ranked in ascending order for 817 RNA-seq gene expression data, the expression levels of ANXA2 gene were defined based on ranking (the top 200 of samples have high expression, and the bottom 200 have low expression). Further data analysis was performed using RStudio (version 3.2.5). Differentially expression genes were screened by *P* < 0.05, log_2_ |Fold Change|> 1 and FDR < 0.05. Enrichment analysis was performed by using the functional annotation chart tool included in DAVID web server 6.8 using as input the list of differentially expressed genes, the enrichment plot was mapped using ImageGP (http://www.ehbio.com/ImageGP/). Venn diagram was mapped using Venny 2.1 (https://bioinfogp.cnb.csic.es/tools/venny/index.html). Five mRNA expression profiles (GSE37751, GSE65194, GSE29044, GSE70947 and GSE29431) are publicly available in the Gene Expression Omnibus (GEO) database.

### Patients’ clinical information

All 194 patients with invasive ductal carcinoma (IDC) were women aged from 31 to 79 year (median age, 52.3 year). During follow-up (median, 68.0 months; range, 2–120 months), 37 (19.1%) patients had recurrence, 27 (13.9%) developed distant metastases (18 cases with bone metastasis, 7 with lung metastasis, 8 with liver metastasis, 3 with brain metastasis). As proposed in the AJCC 8th Ed cancer staging [10], 194 IDC patients were classified into 2 groups (pT1 group and pT2 & pT3 group) according to pathological tumor size (pT).

### Immunohistochemistry analysis and evaluation

Immunohistochemical (IHC) staining was performed as described [11]. In brief, sections (5 μm thick) were dewaxed, hydrated, and heated for antigen retrieval, blocked with hydrogen peroxide and normal goat serum, and subsequently incubated overnight with primary antibody.

IHC score of AQP1, ANXA2 and CTSS were evaluated individually as based on a double scoring system (staining intensity multiplied by staining area). The score of AQP1 was calculated as before [6]. Patients were divided into 3 groups according to IHC score of AQP1: AQP1 score (0), AQP1 score (1–2) and AQP1 score (3–9). Additionally, AQP1 score (0–2) was defined as low expression and AQP1 score (3–9) was defined as high expression.

For the evaluation of cytoplasmic ANXA2 expression, staining intensity was scored as follows: 0 (-) no staining, 1 ( +) definite weak staining, 2 (+ +) intense staining. The staining area was scored as follows: 0 (0–4% of cells stained positive), 1 (5%-24% of cells stained positive), 2 (25%-49% of cells stained positive), 3 (50%-74% of cells stained positive) and 4 (75%-100% of cells stained positive), producing a total range of 0 to 8, cytoplasmic ANXA2 score (0–2) was defined as low expression and AQP1 score (3–8) was defined as high expression. For the evaluation of membrane ANXA2 expression, the cases were divided into membrane positive cases and membrane negative cases.

For the evaluation of CTSS expression, staining intensity was scored as follows: 0 (-) no staining, 1 ( +) definite weak staining, 2 (+ +) moderate staining, 3 (+ + +) intense staining. The staining area was scored as follows: 0 (0–4% of cells stained positive), 1 (5%-30% of cells stained positive), 2 (31%-69% of cells stained positive) and 3 (70%-100% of cells stained positive), producing a total range of 0 to 12, the CTSS score (0–6) was defined as low expression and CTSS score (7–12) was defined as high expression.

### Cell lines and culture conditions and reagents

HEK-293 T cells, MDA-MB-231 cells and Hela cells were cultured in DMEM medium supplemented with 10% fetal bovine serum in a 5% CO_2_ incubator at 37 °C, while T47D cells in 1640 medium. Authentication of the cells was performed by short tandem repeat (STR) analysis in Beijing Microread Genetics Co., Ltd. (Beijing, China). A detailed experimental procedure for primary breast cancer cells was described in the study of Kobayashi et al. [12]. Latrunculin B (SC-203318) was obtained from Santa Cruz Biotechnology. LY3000328 (MCE-HY-15533) was obtained from MedChemExpress.

### Plasmid construction and transfection

To obtain a full-length sequence of AQP1 for functional analysis, one pairs of PCR primers for human AQP1 (GenBank accession No. NM_198098.2, Forward: 5’-AATTGAATTCGCCACCATGGCCAGCGAGTTCAAG-3’ and Reverse: 5’-CGGGATCCCTATTTGGGCTTCATCTC-3’) were utilized for whole genome sequencing as previously described. All over-expression vectors were based on pCDH-CMV-MCS-EF1-Puro lentiviral vector (http://www.addgene.org/). The different tags of AQP1 were cloned into pCDH-CMV-MCS-EF1-Puro lentiviral vector, respectively, such as Flag-AQP1 or GFP-AQP1. The sequences of the inserts were 100% correct. Lentiviral-mediated shRNA knockdown was performed as previously described. Gene-specific shRNAs were introduced into the lentivirus vector plasmid pLKO.1 pure vector. The plasmids were next transfected into HEK-293 T cells with the packing plasmids ΔR and pVSVg to produce lentivirus. Stable lentivirus-infected cells were selected with puromycin or G418 and verified by western blot analysis or RT-qPCR. Plasmids and RNA interference sequences are listed in Supplementary Table S1.

### Western blot analysis and antibodies

In brief, total cell lysates were prepared with cell lysis buffer. After denaturing via boiling, total protein was quantified using a BCA protein assay kit (Pierce). Equal amounts of protein were mixed with 5 × sample buffer and separated by SDS-PAGE and then were transferred onto nitrocellulose membranes (Millipore, Billerica, MA, USA). Primary antibodies were incubated overnight at 4℃, and then membranes were treated with secondary antibodies (IRDye®800CW). Signals were detected using an Odyssey imaging system (Li-Cor Biosciences, Lincoln, NE, USA). Antibodies used in this study were listed in Supplementary Table S2.

### Immunofluorescence assay

Cells were seeded in a 12-well plate, then fixed with 4% paraformaldehyde for 30 min, washed two times in PBS and treated in 0.2% TritonX-100 for 10 min for membrane permeabilization. Blocking was done with 3% BSA for 1 h at room temperature. Primary antibodies were incubated with overnight at 4 °C, and secondary antibodies (Invitrogen, New York, USA) were used at room temperature in dark for 1 h. Cell nuclei were stained with DAPI (Solarbio, Beijing, China), and the cells were photographed with a microscope (Carl Zeiss). The immunofluorescent samples in Figs. 4a and 6e were analyzed using the Zen software (Carl Zeiss Microscopy). Other immunofluorescent images were processed using ImageJ software. The expanding angle of the Golgi apparatus around nuclei was quantified using the ImageJ plug-in ‘Measure’ [13]. All the immunofluorescence and microscopy experiments were performed blinded.

### Fractionation of cytoplasmic proteins

Cytosolic fractions were isolated from the cell pellets using the Nuclear and Cytoplasmic Protein Extraction Kit (P0028, Beyotime, Beijing, China). In brief, cell pellets were resuspended in cytosol extraction reagent (CER) on ice and homogenized in a tight-fitting Dounce homogenizer 50 times and rested on ice for 50 min. The whole cell lysates were then centrifuged at 800 g for 5 min at 4 °C. The supernatant was centrifuged at 4000 g for 5 min at 4 °C three times, collected as cytoplasmic fraction. Cytoplasmic protein fractions were analyzed by Western blot.

### Migration and invasion assays

Migration and invasion abilities of tumor cells were quantitated by using 24 well-plates (Corning, NY, USA) with polycarbonate membranes (8 µm pore size). 8 µm pore size of noncoated (for migration assay) or matrigel-coated (for invasion assay) transwells were used as described by the manufacturer (BD Biosciences). Cells (3 × 10^4^) in DMEM medium were added to the upper chamber, and DMEM culture medium containing 5% fetal bovine serum was added to the lower chamber. Cells were incubated for 16 h, using a cotton swab to remove the cells in the upper chamber, and the invading cells were fixed for 30 min and stained with Giemsa solution for 40 min and photographed under a microscope (Olympus, Tokyo, Japan) at × 200 magnification. For invasion assay, transwell was coated with pre-diluted for 4 h, and then cells were added to the upper chamber. The following steps were the same as migration assay.

### Flag immunoprecipitation

10^7^ cells were collected and lysed with IP lysis buffer (containing protease inhibitors cocktail) at 4 °C overnight. Then, cell lysates were centrifuged at 12,000 rpm for 10 min to collect the supernatant. The supernatant was incubated with 25 µl anti-flag M2 affinity gel at 4 °C overnight and eluted with 3 × flag peptides. Finally, protein eluates were then used for analysis by western blot or mass spectrometry.

### Co-immunoprecipitation (CO-IP)

The cells were lysed in IP lysis buffer (containing protease inhibitors cocktail) at 4 °C overnight, and then 500 µg proteins were incubated overnight with indicated antibody or control IgG at 4 °C, on the second day, adding 40 µl protein A beads (sc-2001, Santa Cruz) into the protein-antibody complex overnight at 4 °C and washed to remove nonspecific binding. Finally, the beads were boiling by the addition of 5 × SDS-PAGE sample buffer for 10 min. The expression of target proteins was examined by Western blot.

### Silver staining and mass spectrometry

Silver staining was following the manufacturer’s suggestions (P0017S, Beyotime Biotechnology). The acrylamide gel strips after silver staining were analyzed by LC–MS/MS in Beijing Genomics Institute (Beijing, China).

### High content live cell imaging for motility experiments

The Operetta® High Content Imaging System (PerkinElmer, Inc.) was used for evaluating the cell migration [14–16]. MDA-MB-231 cells stably transfected Flag-vector or Flag-AQP1 were seeded in a 24-well plate (corning) and were starved for 12 h. Later, the cells were cultured in DMEM medium supplemented with 10% fetal bovine serum in Operetta CLS High Content Imaging System at 37 °C with 5% CO_2_ for 6.5 h at imaging intervals of 30 min. Migrating cells were tracked and imaged using automated single cell tracking algorithm of the Harmony high-content imaging and analysis software (PerkinElmer).

### Transmission electron microscopy

10^6^ cells were resuspended with 100% FBS. After centrifugation (1500 rpm 5 min), the pellets were fixed in 2.5% glutaraldehyde for 4 h at 4 °C. Subsequently, cells were washed for three times with PBS and fixed with 1% osmium tetra-oxide for 1 h at 4 °C. After dehydrated with gradient ethanol and incubated with propylene oxide, cells were embedded with the resin. Ultra-thin sections were stained with 2% uranyl acetate and 1% lead citrate and analyzed by electron microscopy (Hitachi HT7800).

### Preparation of supernatant medium

Cells were grown in 6 cm dish to form 80% confluence and the medium was discarded. Then, cells were washed for three times with PBS and added 2 ml DMEM medium without phenol red for 16 h. Later, the supernatant medium was collected. After centrifugation (3000 g, 5 min), rough supernatant medium samples were filtered through a 0.22 µm filter, thus the precious supernatant medium samples were acquired. Supernatant medium in migration and invasion assays was added to the upper chamber, the following steps were the same as migration/invasion assay. Secreted factors in supernatant medium samples were quantified by mass spectrometry and western blot analysis. For both approaches, conditioned medium values were normalized on the basis of cell lysate values using approaches described elsewhere [17].

### VSVG assay

Cells were transiently transfected with EGFP-VSVG (TS045) plasmid. After 24 h, cells were transferred to a restrictive temperature of 40 °C for 24 h and then transferred to the permissive temperature of 32 °C. Then, cells were fixed for 1 h. The cells that VSVG trafficking to the plasma membrane was calculated from 3 independent experiments. Images were taken using a Zeiss LSM 880 confocal microscope.

### Animal experiments

**Fig. 1:**
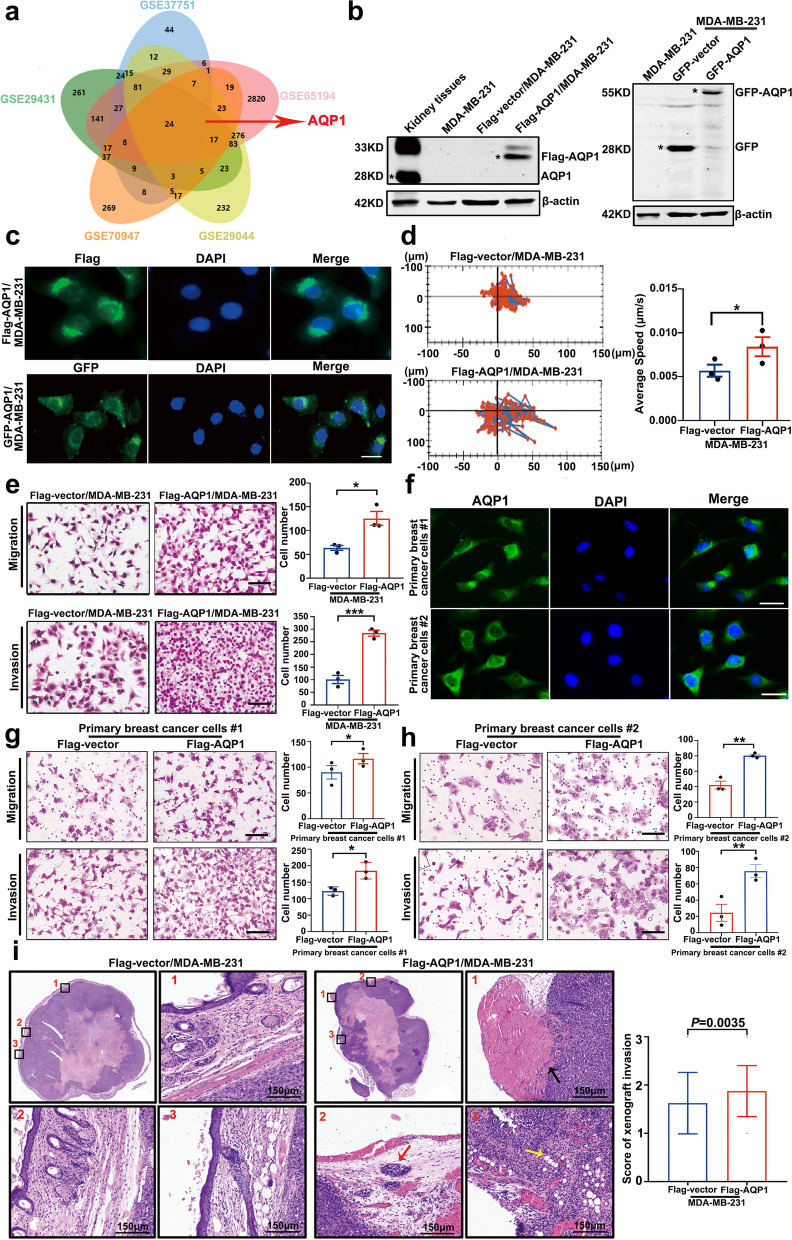
Water channel protein AQP1 mainly localized in the cytoplasm of breast cancer and it was crucial for breast cancer local invasion. **a** A Venn diagram showed several candidate molecules potentially regulating breast cancer progression based on a combination analysis of five gene expression profiles. **b** Western blot analysis of AQP1 expression in kidney tissues and MDA-MB-231 cells with two different tags. Kidney tissues were used as a positive control and β-actin was the loading control. **c** Representative immunofluorescence images of AQP1 localization in MDA-MB-231 cells with two different tags. Scale bar = 20 μm. **d** Migration tracks of MDA-MB-231 cells stably transfected with control (Flag-vector) or AQP1 (Flag-AQP1) and images were captured every 30 min for 6.5 h. Bar graphs show quantification of cell speed (right). Error bars represent SEM of 3 independent experiments (two-tailed Student’s *t* test, ^*^P < 0.05). **e** The abilities of migration and invasion were detected using Flag-vector/MDA-MB-231 and Flag-AQP1/MDA-MB-231 cells. Values were expressed as mean ± SEM from three independent experiments (two-tailed Student’s *t* test, ^*^P < 0.05, ^***^P < 0.001). Scale bar = 100 μm. **f** Immunofluorescence images showed that AQP1 localized in cytoplasm of two primary breast cancer cells. Scale bar = 20 μm. **g**, **h** Representative migration or invasion images of control group and AQP1-overexpressing primary breast cancer cells. Values were expressed as mean ± SEM from three independent experiments (two-tailed Student’s *t* test, ^*^P < 0.05, ^**^P < 0.01). Scale bar = 100 μm. **i** Representative images of hematoxylin–eosin staining in xenograft paraffin specimens, part of square 1/2/3 was enlarged in picture 1/2/3 respectively (muscle involvement: black arrow, Satellite nodules: red arrow, adipocytes involvement: yellow arrow). *N* = 12/group. The score of xenografts invasion in mice was analyzed quantitatively. All in vitro experiments were repeated at least 3 times

All animal work was approved by The Animal Ethical and Welfare Committee of Tianjin Medical University Cancer Institute and Hospital (NSFC-AE-2021113). In Fig. 1i, 5 × 10^6^ Flag-vector/MDA-MB-231 or Flag-AQP1/MDA-MB-231 cells suspended in 200 µl PBS were injected into the third left mammary fat pads of 3–4 weeks old female BALB/c nude mice (Model Animal Research Center of Nanjing University, Nanjing, China). When the volume of the tumor was enough to be inoculated, Flag-vector/MDA-MB-231 or Flag-AQP1/MDA-MB-231 tumors were randomly chosen and cut into small pieces (2 × 2 mm) and subsequently anchored to the third pair of fat pads on the right side of new nude mice (Mice were randomly assigned into two groups, *n* = 12/group). Tumor size was measured by using a caliper every 4 days. The tumor volume was calculated as V = ab^2^/2 (a: larger diameter; b: shorter diameter). After a month, the mice were sacrificed and the tumor were removed and fixed with 4% paraformaldehyde. Samples were soaked in wax and then were cut to 5-mm-thick sections with routine histological methods. HE staining was performed to observe the invasion of the xenograft tumors. The score of xenografts invasion in mice was the sum of muscle involvement, adipocytes involvement and satellite nodules. The expression of Ki67 in xenograft tumors was measured by IHC assay.

In Fig. 8a-e, 5 × 10^6^ Flag-vector/MDA-MB-231, Flag-AQP1/MDA-MB-231, shANXA2 #1/MDA-MB-231, Flag-AQP1/shANXA2 #1/MDA-MB-231, shRab1b #1/MDA-MB-231 or Flag-AQP1/shRab1b #1/MDA-MB-231 cells suspended in 200 µl PBS were injected into the third left mammary fat pads of 3–4 weeks old female BALB/c nude mice (Model Animal Research Center of Nanjing University, Nanjing, China). When the volume of the tumor was enough to be inoculated, tumors were randomly chosen and cut into small pieces (2 × 2 mm) and subsequently anchored to the third pair of fat pads on the right side of new nude mice. Six different groups (*n* = 14–20/group) were set up. Total of the mice were monitored for survival.

### F-actin staining

Briefly, after starving 24 h, indicated cells were treated with DMSO or 0.1 µM latrunculin B for 1 h. Then cells were fixed with 4% paraformaldehyde and were incubated with Alexa Fluor 488-phalloidin (1:500 dilution) for 1 h at room temperature. The nuclei were further stained with 4’, 6- diamidino-2-phenylindole (DAPI) for 10 min. Images were taken using a Zeiss LSM 880 confocal microscope.

### Statistical analysis

The SPSS 19.0 software package (SPSS, Chicago, IL, USA) and GraphPad Prism were used for statistical analysis. Chi-square test was performed for group comparisons. The Cox proportional hazards regression model was performed toward the identification of relevant prognostic factors. Survival curves were plotted by the Kaplan–Meier method and compared by the log-rank test. For in vitro work, statistical significance for comparisons between two different groups was determined using a two-tailed Student’s *t* test. All in vitro experiments were repeated at least 3 or 4 times. *P* < 0.05 was considered statistically significant in all analyses.

## Results

### Water channel protein AQP1 mainly localized in the cytoplasm of breast cancer and it was crucial for breast cancer local invasion

In order to identify important molecules that potentially participate in breast cancer progression, we applied five mRNA expression profiles (GSE37751, GSE65194, GSE29044, GSE70947, and GSE29431) to generate specific sets of genome-wide genes in breast cancer tissues compared with normal tissues. The Venn diagram showed 24 differentially expressed genes that included AQP1 (Fig. 1a).

We performed immunohistochemistry analysis in 194 cases of invasive ductal carcinoma (IDC), and found that high cytoplasmic expression of AQP1 was positively associated with metastasis or recurrence (*P* = 0.009), lymph node metastasis (*P* = 0.038), tumor size (*P* = 0.003), and pTNM stage (*P* = 0.003) (Supplementary Fig. 1a, b and Supplementary Table S3). High cytoplasmic expression of AQP1 was found to be positively associated with pathological tumor size (pT) (Supplementary Fig. 1c, d and Supplementary Table S4). Using multivariate Cox regression analysis, high expression of AQP1 was proved to be an independent prognostic factor for overall survival (OS) (*P* = 0.027) and progression-free survival (PFS) (*P* = 0.027) (Supplementary Table S5).

We constructed stably transduced MDA-MB-231 cell lines (which do not express endogenous AQP1 [18]) over-expressing Flag-tagged or GFP-tagged AQP1, designated as Flag-AQP1/MDA-MB-231 and GFP-AQP1/MDA-MB-231, respectively. Flag-vector/MDA-MB-231 and GFP-vector/MDA-MB-231 were used as negative controls (Fig. 1b). In MDA-MB-231 cells expressing Flag- and GFP-tagged AQP1, AQP1 had a cytoplasmic localization found primarily around the nucleus (Fig. 1c).

We tested the effects of AQP1-overexpression on breast cancer biological function. Compared with the control group, AQP1-overexpressing cells exhibited increased cell movement by cell tracker assay (*P* = 0.0282, Fig. 1d). The migration and invasion assays indicated that AQP1 overexpression promoted the migration and invasion abilities of MDA-MB-231 cells (*P* = 0.0163 for migration, *P* = 0.0007 for invasion, Fig. 1e).

Flag-AQP1/MDA-MB-231 cells expressing low levels of AQP1 were constructed (designed as Flag-AQP1/shAQP1/MDA-MB-231) (Supplementary Fig. 2a). Migration and invasion assays showed that AQP1 knockdown decreased cell migration and invasion abilities (*P* = 0.0019 for migration, *P* = 0.0053 for invasion, Supplementary Fig. 2b, c). Stably transduced cell lines over-expressing Flag-tagged AQP1 were constructed in T47D cells and designated as Flag-AQP1/T47D (Supplementary Fig. 3a). Invasion assays showed that Flag-AQP1/T47D cells had higher invasion abilities than the control cells (Flag-vector/T47D) (*P* = 0.0087, Supplementary Fig. 3b).

Since endogenous AQP1 was undetectable in MDA-MB-231 cell lines, we utilized two primary breast cancer cell lines to investigate the function of AQP1. Cytoplasmic staining of AQP1 was confirmed in two primary breast cancer cell lines, with localization consistent with AQP1 localization in the clinical samples and AQP1-overexpressing MDA-MB-231 cells (Fig. 1f). Functional cell experiments showed that the two primary breast cancer cell lines over-expressing AQP1 demonstrated increased migration and invasion abilities (Fig. 1g, h). We further validated the function of AQP1 in breast cancer local invasion in vivo using xenograft mouse model. The purpose of the experiment was to detect the local invasion of xenograft tumors. The end time point of the two groups was one month after transplantation. HE staining was performed to observe the invasion of the xenograft tumors. In details, Flag-vector/MDA-MB-231 or Flag-AQP1/MDA-MB-231 xenograft tumors (which were described in materials and methods) were cut into small pieces (2 × 2 mm) and subsequently anchored to the third pair of fat pads on the right side of new nude mice (Mice were randomly assigned into two groups, *n* = 12/group). Tumor size was measured by using a caliper every 4 days. The tumor volume was calculated as V = ab^2^/2 (a: larger diameter; b: shorter diameter). After a month, the mice were sacrificed and the tumors were removed and fixed with 4% paraformaldehyde. Samples were soaked in wax and then were cut to 5-mm-thick sections with routine histological methods. HE staining was performed on the xenograft tumors to observe the local invasion. The score of xenografts invasion in mice was the sum of muscle involvement, adipocytes involvement and satellite nodules. Notably, AQP1 overexpression promoted the local invasion of the experimental group compared with the control (*P* = 0.0035, Fig. 1i). Compared with control group, the tumor volume from AQP1 overexpression mice group was remarkably increased (Supplementary Fig. 1e). Furthermore, we examined the expression of Ki-67 in the xenograft tumors by IHC which demonstrated that Ki-67 positive rate was higher (^*^P < 0.05) in the AQP1 overexpression tumor (34.25% ± 14.23%) compared with the control tumor (48.08% ± 11.30%) as shown in the Supplementary Fig. 1f. These experiments supported that AQP1 had a primarily cytoplasmic location in breast cancer cells and that a high cytoplasmic expression of AQP1 contributed to breast cancer local invasion in vitro and in vivo.

### AQP1 localized in the Golgi apparatus and induced cell secretion of ICAM1 and CTSS, leading to the cell local invasion

We used RNA-sequencing data to further explore how AQP1 modulated breast cancer local invasion. RNA-seq data for 817 breast cancer patients were downloaded from cBioportal (http://www.cbioportal.org/). The gene expression profile was divided into two groups according to the level of AQP1. We found 1805 differentially expressed genes (using the criteria of *P* < 0.05, log_2_ |fold change|> 1, and FDR < 0.05). According to the “up_keywords” in the online DAVID tool (https://david.ncifcrf.gov/home.jsp), we found many differentially expressed genes related to the category of “secretion” (Supplementary Fig. 4a). GO enrichment and KEGG pathway analyses of these 1805 differential genes were carried out. Significant enrichment of biological process terms (*P* < 0.05) revealed that genes associated with AQP1 were involved primarily in extracellular matrix disassembly and extracellular matrix organization (Supplementary Fig. 4b). Molecular functions enriched for AQP1-related genes were primarily related to extracellular matrix binding and collagen binding (Supplementary Fig. 4c). GO classification of the AQP1-related genes revealed that proteinaceous extracellular matrix, extracellular matrix, and Golgi lumen were enriched in GO terms of cellular component (Supplementary Fig. 4d). KEGG analysis of AQP1-related genes showed enrichment in extracellular matrix-receptor interaction and cell adhesion molecules (CAMs) (Supplementary Fig. 4e).

**Fig. 2:**
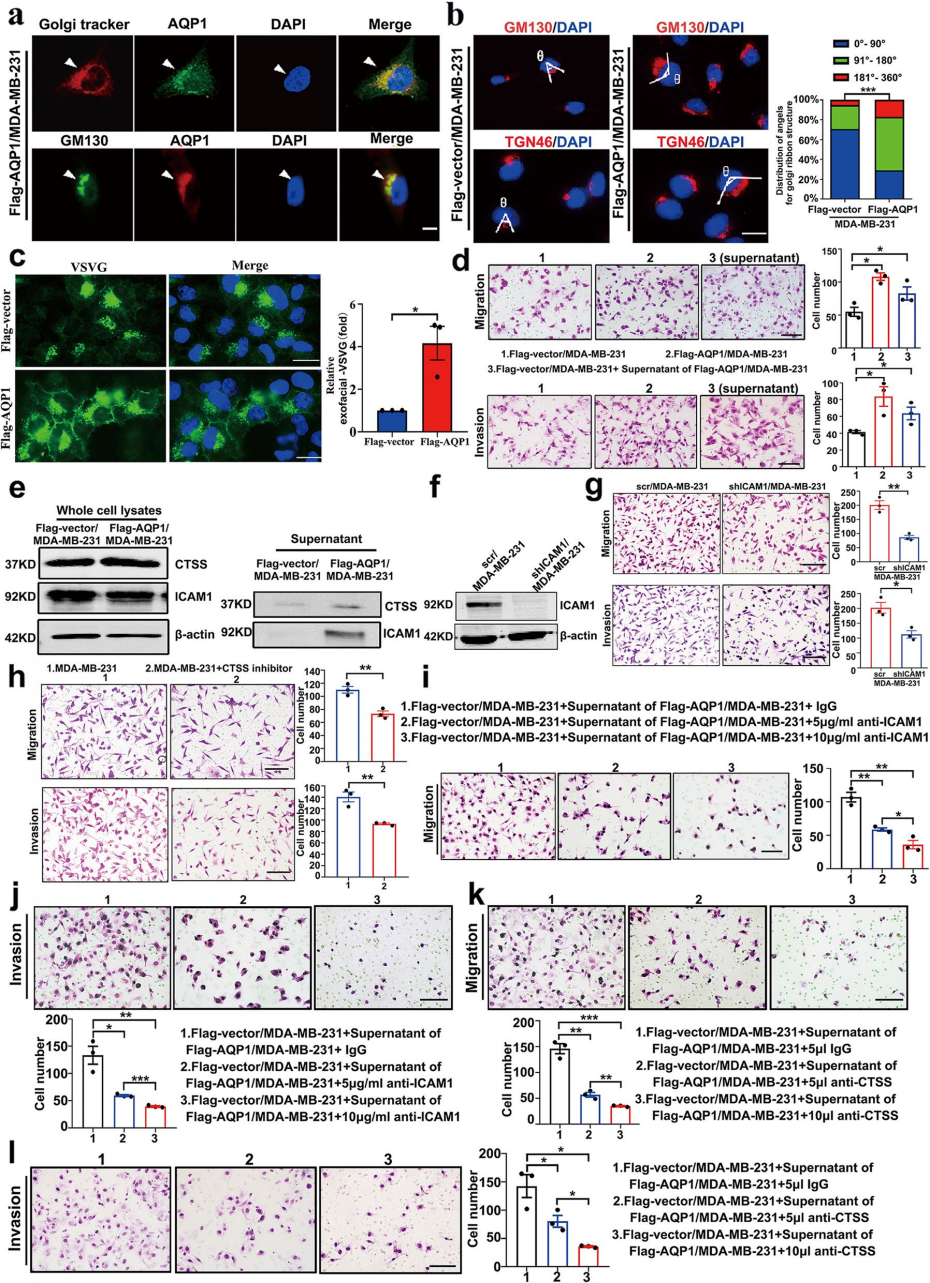
AQP1 localized in the Golgi apparatus and induced cell secretion of ICAM1 and CTSS, leading to the cell local invasion. **a** Immunofluorescent staining of AQP1 and Golgi marker GM130 or Golgi tracker in Flag-AQP1/MDA-MB-231 cells. Scale bar = 20 μm. **b** Immunofluorescent staining of GM130/TGN46 together with staining of DAPI in Flag-vector/MDA-MB-231 and Flag-AQP1/MDA-MB-231 cells. In the right panel showed the distribution of cells with different ranges of Golgi ribbon angle (Ɵ: 0°–360°) (*n* = 80–90 cells per group, ^***^P < 0.001). Scale bar = 20 μm. **c** Trafficking of TS045-VSVG-EGFP in Flag-vector/hela and Flag-AQP1/hela cells (two-tailed Student’s *t* test, ^*^P < 0.05). Scale bar = 20 μm. **d** Migration and invasion assays showed that Flag-vector/MDA-MB-231 cells treated with the supernatant of Flag-AQP1/MDA-MB-231 cells exhibited the promoted phenotype compared with Flag-vector/MDA-MB-231 cells (two-tailed Student’s *t* test, ^*^P < 0.05). Each bar represented the mean ± SEM from three independent experiments. Scale bar = 100 µm. **e** Western blot analysis to detect the expression of CTSS and ICAM1 in the whole cell lysates and supernatant medium of Flag-vector/MDA-MB-231 cells and Flag-AQP1/MDA-MB-231 cells. β-actin was the loading control. **f** Western blot of ICAM1 expression in control and ICAM1-downexpressing cells. **g** Representative migration or invasion images of control and ICAM1-downexpressing groups (two-tailed Student’s* t* test, ^*^P < 0.05, ^**^P < 0.01). Scale bar = 100 µm. **h** Representative migration or invasion images of control and CTSS inhibition groups (two-tailed Student’s *t* test, ^**^P < 0.01). Scale bar = 100 µm. **i**, **j** Representative migration or invasion images from Flag-vector/MDA-MB-231 cells treated with the supernatant medium samples plus 5 µg/ml or 10 µg/ml ICAM1 neutralizing antibody (anti-ICAM1) or control IgG (two-tailed Student’s *t* test, ^*^P < 0.05, ^**^P < 0.01, ^***^P < 0.001). Each bar represented the mean ± SEM from three independent experiments. Scale bar = 100 µm. **k**, **l** Representative migration or invasion images from Flag-vector/MDA-MB-231 cells treated with the supernatant medium samples plus 5 µl or 10 µl CTSS neutralizing antibody (anti-CTSS) or control IgG (two-tailed Student’s *t* test, ^*^P < 0.05, ^**^P < 0.01, ^***^P < 0.001). Each bar represented the mean ± SEM from three independent experiments. Scale bar = 100 µm. All in vitro experiments were repeated at least 3 or 4 times

The Golgi apparatus is a cellular organelle that plays a critical role in the processing of proteins for secretion. Extended Golgi apparatus morphology is a key morphological feature of highly metastatic cells [19, 20]. We noticed that both Flag- and GFP-tagged AQP1 MDA-MB-231 cells displayed diffuse cytoplasmic localization as previously described in Fig. 1c, but showed the most intense staining in the perinuclear regions that were co-stained with a Golgi apparatus tracker and the GM130 marker of the cis-Golgi network (Fig. 2a). Immunofluorescent staining of the Golgi apparatus marker (anti-GM130 antibody and anti-TGN46 antibody), we found that AQP1-overexpressing cells exhibited more extended distribution of perinuclear Golgi apparatus staining (^***^P < 0.001, Fig. 2b), and the extent of spreading, as measured by the angle of spreading in individual cells [13, 21]. We defined spreading as an angle of 91°-180°. Furthermore, transmission electron microscopy (TEM) observations clearly demonstrated that AQP1-overexpressing cells presented the more extend Golgi compared with control cells (Supplementary Fig. 5a). To find out whether the extended morphology of Golgi apparatus induced by upregulated AQP1 affected the Golgi apparatus secretory capacity, we transfected cells with a temperature-sensitive mutant vesicular stomatitis virus (VSVG) that could accumulate in the Golgi apparatus at a restrictive temperature and could be released for anterograde trafficking at a permissive temperature [22]. Over-expressing AQP1 cells were constructed in Hela cells (Supplementary Fig. 6a). Compared to the control group, AQP1-overexpressing cells accumulated more VSVG on the plasma membrane (*P* = 0.0162, ^*^P < 0.05, Fig. 2c). Then we treated Flag-vector/MDA-MB-231 cells with the supernatant of AQP1-overexpressing cells and conducted migration and invasion experiments. Compared with the control group, cells treated with the supernatant of AQP1-overexpressing cells showed increased capabilities of migration and invasion (^*^P < 0.05, Fig. 2d). However, the supernatant from downregulated AQP1 in AQP1-overexpressing cells decreased the cell migration and invasion abilities (*P* = 0.0436 for migration, *P* = 0.0002 for invasion, ^*^P < 0.05, ^***^P < 0.001, Supplementary Fig. 2d, e). Flag-vector/T47D cells treated with the supernatant medium of Flag-AQP1/T47D cells also exhibited increased invasion abilities (^*^P < 0.05, Supplementary Fig. 3c). These results suggested that over-expressing AQP1 promoted cell secretion of prometastatic proteins.

We sought to identify AQP1-dependent secreted proteins that induced breast cancer cell local invasion. We collected cell culture supernatants from the control group and AQP1-overexpressing cells and performed mass spectrometry analysis. Proteins detected in the supernatants of Flag-vector/MDA-MB-231 cells and Flag-AQP1/MDA-MB-231 cells are listed in Supplementary Tables S6 and S7. Mass spectrometry analysis on supernatant medium samples identified approximately 200 proteins in each group. There were 76 proteins detected only in the AQP1-overexpressing group, including proteins reported to exert prosurvival and/or prometastatic activities in breast cancer, such as intercellular adhesion molecule 1 (ICAM1) [23, 24], cathepsin S (CTSS) [25, 26], and PLAT [27] (Supplementary Fig. 5b). GO biological process analysis indicated that these proteins might regulate extracellular matrix disassembly (Supplementary Fig. 5c). GO molecular function analysis showed that the 76 proteins from the AQP1-overexpressing group were enriched in “collagen and fibronectin binding” part (Supplementary Fig. 5d). 17 from 76 proteins in AQP1-overexpressing group were mainly located in the extracellular space and extracellular matrix (Supplementary Fig. 5e).

We randomly selected RNA-seq data of 15 cases with AQP1 high expression and 15 cases with AQP1 low expression from the 817 breast cancer patients to characterize the similarity between AQP1 and other 17 proteins in the two RNA-seq groups. Using a hierarchical cluster analysis heatmap (distance measure using Pearson), we found that ICAM1 and CTSS showed high expression similarities with AQP1 (Supplementary Fig. 5f). It has been reported that ICAM1 is a promising TNBC target and biomarker. Immunotargeting of ICAM1 may be a strategy for imaging and treatment of TNBC, as the ICAM1 antibody reduces TNBC cell migration [24]. Similarly, CTSS was previously reported in cancer metastasis for its role of degrading extracellular matrix (ECM) proteins including laminin, fibronectin elastin, osteocalcin and some collagens [28]. Further western blot assays showed that total protein expression levels of CTSS and ICAM1 in whole-cell lysates remained unchanged, but the supernatant medium expression of CTSS and ICAM1 was increased in the AQP1-overexpressing group (Fig. 2e).

We constructed a stably-transduced cell line expressing a low level of ICAM1, designed as shICAM1/MDA-MB-231 and scr/MDA-MB-231 cells were regarded as a negative control (Fig. 2f). Downregulation of ICAM1 via shRNA knockdown decreased cell migration and invasion abilities (*P* = 0.0024 for migration, *P* = 0.0150 for invasion, ^**^P < 0.01, ^*^P < 0.05, Fig. 2g). CTSS is a kind of cysteine protease and LY3000328 is a potent and specific inhibitor of CTSS [29, 30]. After treatment with the CTSS inhibitor LY3000328, the cell migration and invasion abilities also decreased compared with control cells (*P* = 0.0054 for migration, *P* = 0.0058 for invasion, ^**^P < 0.01, Fig. 2h). The abilities of migration and invasion were decreased with antibody dose in Flag-vector/MDA-MB-231 cells, when treated with supernatant medium of Flag-AQP1/MDA-MB-231 cells plus with 5 µg/ml or 10 µg/ml ICAM1 antibody or control IgG (Fig. 2i, j). Similar results were observed after treating Flag-vector/MDA-MB-231 cells with supernatant medium of Flag-AQP1/MDA-MB-231 cells plus with 5 µl/10 µl CTSS antibody or control IgG (Fig. 2k, l). Thus, we proposed that AQP1 promoted local invasion through augmenting the cellular release of pro-metastatic proteins ICAM1 and CTSS.

### AQP1 interacted with ANXA2 and recruited it from the cell membrane to the cytoplasm

**Fig. 3:**
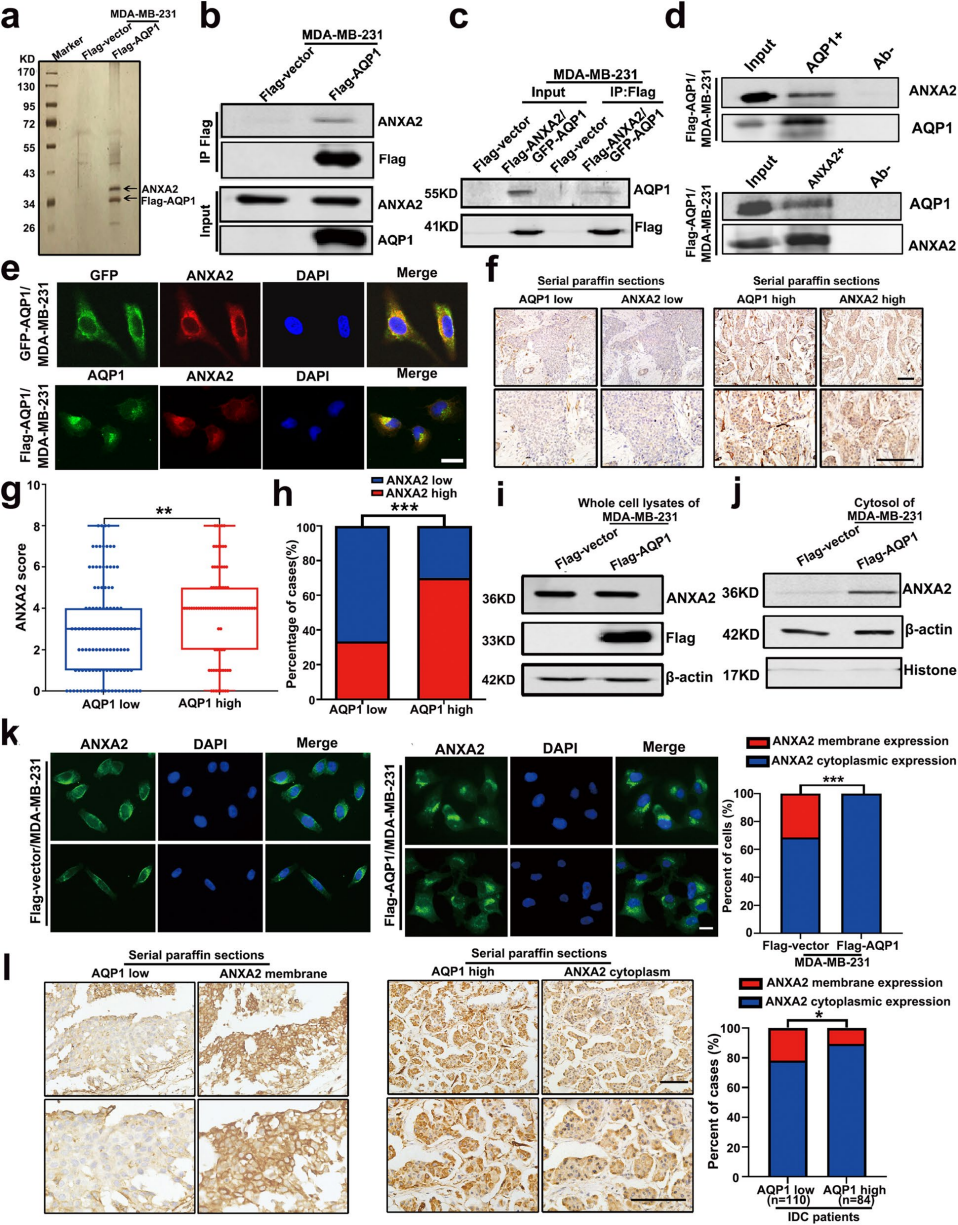
AQP1 interacted with ANXA2 and recruited it from the cell membrane to the cytoplasm. **a** Cellular extracts from MDA-MB-231 cells stably transfected Flag-vector or Flag-AQP1 were immunopurified with anti-Flag affinity beads and eluted with Flag peptides. The eluates were resolved on SDS-PAGE and silver-stained followed by mass spectrometry analysis. **b**-**c** Immunoprecipitation experiments were performed by using an anti-flag M2 affinity gel and then immunoblotted with ANXA2 or AQP1 antibodies. **d** Whole cell lysates from Flag-AQP1/MDA-MB-231 cells were immunoprecipitated with anti-ANXA2 or anti-AQP1 and then immunoblotted with antibodies against the indicated proteins. **e** Immunofluorescence analysis showed the colocalization of AQP1 and ANXA2 in GFP-AQP1/MDA-MB-231 and Flag-AQP1/MDA-MB-231 cells. Scale bar = 20 μm. **f** Immunohistochemical staining of 194 serial paraffin sections was performed to detect the expression of AQP1 and ANXA2. Scale bar = 100 μm. **g** Cytoplasmic ANXA2 score in AQP1 high expression group was higher than in AQP1 low expression group in 194 serial paraffin sections (two-tailed Student’s *t* test, *P* = 0.0034, ^**^P < 0.01). **h** 70.2% (59/84) IDC patients exhibited high ANXA2 cytoplasmic expression in AQP1 high cytoplasmic expression group, while the percent of high ANXA2 cytoplasmic expression cases was only 33.6% (37/110) in AQP1 low cytoplasmic expression group (Chi-square test,* P* = 0.000, ^***^P < 0.001). **i** Western blot analysis of ANXA2 expression in Flag-vector/MDA-MB-231 and Flag-AQP1/MDA-MB-231 cells. **j** Western blot analysis of cytoplasmic ANXA2 expression in the cytosol of Flag-vector/MDA-MB-231 and Flag-AQP1/MDA-MB-231 cells. **k** Immunofluorescent assay showed the cellular location of ANXA2 in Flag-vector/MDA-MB-231 and Flag-AQP1/MDA-MB-231 cells (*n* = 194–197 cells per group, Chi-square test, ^***^P < 0.001). Scale bar = 20 μm. **l** Immunohistochemical staining of 194 serial paraffin sections was performed to detect the expression of AQP1 and ANXA2. 21.8% (24/110) IDC patients exhibited ANXA2 membrane expression in AQP1 low cytoplasmic expression group, while the percent of ANXA2 membrane expression cases was only 10.7% (9/84) in AQP1 high cytoplasmic expression group (Chi-square test, *P* = 0.041, ^*^P < 0.05). Scale bar = 100 μm. All in vitro experiments were repeated at least 3 times

To clarify further mechanistic insights into the cellular function of AQP1 in breast cancer local invasion, we sought to identify additional potential AQP1-interacting proteins. We performed Flag immunoprecipitation (IP) in AQP1 expression cells, and the products were silver-stained and assessed using mass spectrometry quantification (Fig. 3a). In addition to Keratin, ANXA2 scored highest in the mass spectrometry results. We constructed stably transfected Flag-tagged ANXA2 in over-expressing GFP-AQP1/MDA-MB-231 cells (Supplementary Fig. 6b). Immunoprecipitation experiments showed that AQP1 is bound to ANXA2, a phospholipid-binding protein in a calcium-dependent manner [31–33] (Fig. 3b-d). To further elucidate the cellular function of the interaction between AQP1 and ANXA2, we performed colocalization experiments and found that the vast majority of the overlap between AQP1 and ANXA2 was detected in the perinuclear Golgi region, whether in MDA-MB-231 cells or in T47D cells (Fig. 3e and Supplementary Fig. 3d).

We performed serial sections in 194 IDC patients’ tissues and analyzed the correlation between the expression patterns of AQP1 and ANXA2 by histochemical staining. The results showed that there was a positive correlation between the expression of AQP1 and ANXA2 (Fig. 3f-h and Supplementary Table S8, *r*_*s*_ = 0.363). In Fig. 3 h, 70.2% (59/84) IDC patients exhibited high ANXA2 cytoplasmic expression in AQP1 high cytoplasmic expression group, while the percent of high ANXA2 cytoplasmic expression cases was only 33.6% (37/110) in AQP1 low cytoplasmic expression group (Chi-square test,* P* = 0.000, ^***^P < 0.001). Western blot results for whole-cell lysates of Flag-vector/MDA-MB-231 and Flag-AQP1/MDA-MB-231 showed that over-expressing AQP1 had no effect on ANXA2 expression (Fig. 3i). However, AQP1 overexpression promoted cytosolic localization of ANXA2 (Fig. 3j). Meanwhile, immunofluorescence assays showed that ANXA2 was primarily located in the cytosol and membrane in Flag-vector/MDA-MB-231 cells, but in the presence of overexpressed AQP1, almost all ANXA2 displaced cytosolic positioning (Chi-square test,* P* = 0.000, ^***^P < 0.001, Fig. 3k).

Immunohistochemical analysis of ANXA2 was performed in the samples from the 194 IDC patients. Among the 110 low-cytoplasmic-AQP1-expressing samples, 21.8% (24/110) of breast cancer tissues showed positive ANXA2 membranous expression. Among the 84 high-cytoplasmic-AQP1-expressing samples, 10.7% (9/84) showed positive ANXA2 membranous expression (Supplementary Table S9). Tissues from patients with high expression of AQP1 showed a lower membranous location of ANXA2 (Chi-square test, *P* = 0.041, ^*^P < 0.05, Fig. 3l). These results showed that AQP1 interacted with ANXA2 and recruited it from the cell membrane to the cytoplasm.

### AQP1 recruited ANXA2 to the Golgi and promoted the Golgi extension through F-actin by interaction with ANXA2, inducing breast cancer cell invasion

**Fig. 4:**
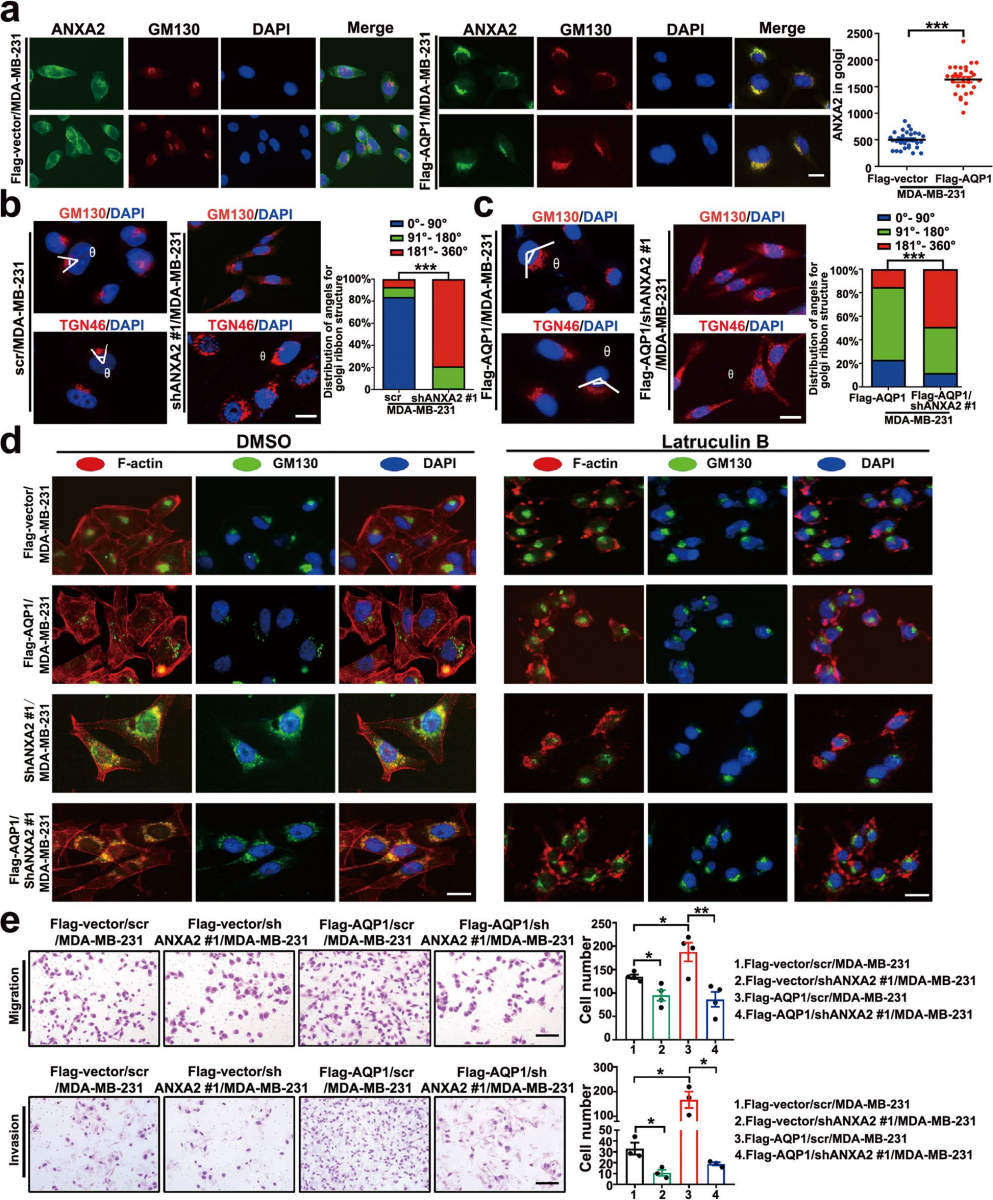
AQP1 recruited ANXA2 to the Golgi apparatus and promoted the Golgi extension through F-actin by interaction with ANXA2, inducing breast cancer cell invasion. **a** Immunofluorescent assay showed the colocalization of ANXA2 and GM130 in Flag-vector/MDA-MB-231 and Flag-AQP1/MDA-MB-231 cells (*n* = 30 cells per group, two-tailed Student’s *t* test, ^***^P < 0.001). Scale bar = 20 μm. **b**-**c** Immunofluorescent staining of GM130/TGN46 and DAPI in 4 different cell clones. In the right panel showed the distribution of cells with different ranges of Golgi ribbon angle (Ɵ: 0°–360°), respectively (*n* = 74–97 cells per group, ^***^P < 0.001). Scale bar = 20 μm. **d** Flag-AQP1/MDA-MB-231 cells showed extended Golgi morphology (GM130, green) and normal F-actin (Texas red phalloidin). When treated with latrunculin B, Flag-AQP1/MDA-MB-231 cells exhibited loss of F-actin (stress fibers and peripheral actin) and Golgi condensation. Flag-AQP1/shANXA2 #1/MDA-MB-231 cells also reversed the extended Golgi morphology when treatment with latrunculin B. Scale bar = 20 μm. **e** The abilities of migration and invasion were detected using four indicated cells. Each bar represented the mean ± SEM from three or four independent experiments (two-tailed Student’s *t* test, ^*^P < 0.05, ^**^P < 0.01). Scale bar = 100 µm. All in vitro experiments were repeated at least 3 or 4 times

To determine the effect of the AQP1 on the Golgi localization of ANXA2, we performed immunofluorescence staining in the control group and AQP1-overexpressing cells. The results showed that over-expressing AQP1 increased the Golgi apparatus location of ANXA2 (^***^P < 0.001, Fig. 4a). ANXA2 expression was knocked down by 3 different shRNAs in MDA-MB-231 cells. ANXA2 expression decreased in shANXA2 #1/MDA-MB-231 cells (Supplementary Fig. 6c). We stably transfected shANXA2 #1 into Flag-vector/MDA-MB-231 cells and Flag-AQP1/MDA-MB-231 cells (Supplementary Fig. 6d). We investigated whether ANXA2 influenced Golgi apparatus morphology. Cell lines stably over-expressing ANXA2 were constructed by lentiviral transduction (Supplementary Fig. 6e). Cells over-expressing ANXA2 (Flag-ANXA2/MDA-MB-231) exhibited an extended Golgi apparatus phenotype (Supplementary Fig. 6f). Cells with downregulated ANXA2 (shANXA2 #1/MDA-MB-231) caused Golgi apparatus fragmentation (Fig. 4b), which is related to reduced secretion of cancer-promoting growth factors [34]. AQP1-mediated Golgi apparatus extension was dependent on the expression of ANXA2 in the Golgi apparatus, as the downregulated expression of ANXA2 reversed the extended Golgi apparatus phenotype induced by AQP1-overexpression (Fig. 4c).

Actin filaments are involved in the maintenance of Golgi apparatus morphology [35, 36] and ANXA2 is an F-actin binding protein, which regulates actin cytoskeleton dynamics [37, 38]. We used an actin polymerization inhibitor latrunculin B to perturb the actin cytoskeleton in indicated cell lines. When the AQP1-overexpressing cells were treated with the F-actin inhibitor latrunculin B, the extented phenotype of Golgi phenotype was reversed (Fig. 4d). Similar effects were also observed in ANXA2-knockdown cells and ANXA2-knockdown AQP1-overexpressing cells, for which latrunculin B treatment reversed shANXA2-induced Golgi dispersion and resulted in the formation of perinuclear Golgi clusters (Fig. 4d). The above results showed that AQP1-induced Golgi apparatus extension was ANXA2/F-actin dependent. Migration and invasion assays showed that reduction of ANXA2 abrogated the AQP1-mediated enhancement of migration and invasion capacities (Fig. 4e). Collectively, these findings revealed that AQP1 interacted with ANXA2 to promote Golgi apparatus extension through F-actin, inducing breast cancer cell invasion.

### The interaction of the NT-6 × helix domain of AQP1 with ANXA2 induced cell secretion of ICAM1 and CTSS that led to cell local invasion

**Fig. 5:**
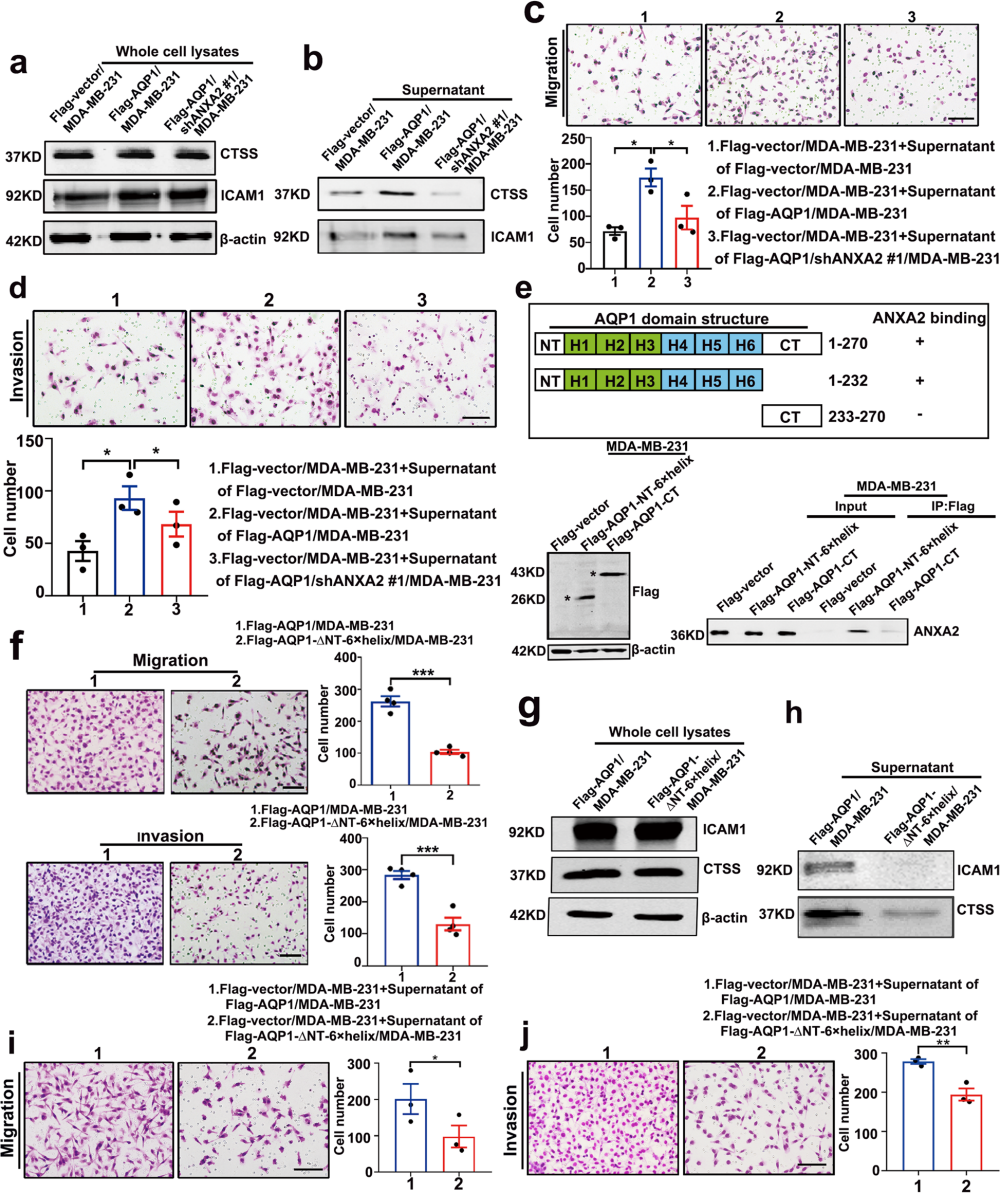
The interaction of the NT-6 × helix domain of AQP1 with ANXA2 induced cell secretion of ICAM1 and CTSS, leading to cell local invasion. **a** Western blot analysis to detect the expression of CTSS and ICAM1 in the whole cell lysates of Flag-vector/MDA-MB-231 cells, Flag-AQP1/MDA-MB-231 cells and Flag-AQP1/shANXA2 #1/MDA-MB-231 cells. β-actin was the loading control. **b** Western blot analysis of the supernatant medium samples isolated from 3 indicated cells was performed to quantify ICAM1 and CTSS proteins. **c**-**d** Representative migration or invasion images from Flag-vector/MDA-MB-231 cells treated with 3 different supernatant medium samples (two-tailed Student’s *t* test, ^*^P < 0.05). Each bar represented the mean ± SEM from three independent experiments. Scale bar = 100 µm. **e** Co-immunoprecipitation experiments were performed to illustrated the interaction between AQP1-NT-6 × helix and ANXA2. The Flag-AQP1-CT/MDA-MB-231 cells were also tagged with GFP. **f** The abilities of migration and invasion were detected using Flag-AQP1/MDA-MB-231 and Flag-AQP1-∆NT-6 × helix/MDA-MB-231 cells. Each bar represented the mean ± SEM from four independent experiments (two-tailed Student’s *t* test, ^***^P < 0.001). Scale bar = 100 µm. **g** Western blot analysis to detect the expression of CTSS and ICAM1 in the whole cell lysates of Flag-AQP1/MDA-MB-231 cells and Flag-AQP1-∆NT-6 × helix/MDA-MB-231 cells. β-actin was the loading control. **h** Western blot analysis of the supernatant medium samples in 2 indicated cells. **i**-**j** Representative migration or invasion images from Flag-vector/MDA-MB-231 cells treated with the supernatant medium of Flag-AQP1/MDA-MB-231 cells and that of Flag-AQP1-∆NT-6 × helix/MDA-MB-231 cells (two-tailed Student’s *t* test, ^*^P < 0.05, ^**^P < 0.01). Each bar represented the mean ± SEM from three independent experiments. Scale bar = 100 µm. All in vitro experiments were repeated at least 3 or 4 times

To determine the effect of ANXA2 on the secretion of ICAM1 and CTSS, we performed western blot assays, the results showed that increased secretion of ICAM1 and CTSS in cells over-expressing AQP1 was ANXA2-dependent, as the total protein levels of ICAM1 and CTSS were unchanged in whole-cell lysates (Fig. 5a), but the expression levels of ICAM1 and CTSS were decreased in the supernatant medium collected from AQP1-overexpressing cells where ANXA2 was knocked down compared with the supernatant medium collected from AQP1-overexpressing cells (Fig. 5b). Increased migration and invasion abilities induced by the addition of supernatant medium from AQP1-overexpressing were reversed by the addition of supernatant medium from AQP1-overexpressing, ANXA2 knocked down cells (Fig. 5c, d).

According to the previous study [18], we divided AQP1 into two domains (the NT-6 × helix and CT domains) and established MDA-MB-231 cell lines stably expressing different domains of AQP1, which were designated as Flag-AQP1-NT-6 × helix/MDA-MB-231 and Flag-AQP1-CT/MDA-MB-231 (Fig. 5e-left). Immunoprecipitation assays showed that ANXA2 was bound to the NT-6 × helix domain of AQP1 (Fig. 5e-right). Cells deleted NT-6 × helix domain of AQP1 were designated as Flag-AQP1-delete-NT-6 × helix/MDA-MB-231. We next asked if binding to ANXA2 was required for the pro-metastatic function of AQP1 by performing migration and invasion assays. We found that cell migration and invasion were reduced in cells over-expressing AQP1-delete-NT-6 × helix compared with AQP1 over-expressing cells (*P* < 0.001 for migration, *P* = 0.0006 for invasion, two-tailed Student’s *t* test, ^***^P < 0.001, Fig. 5f). Western blot assays showed that over-expressing AQP1-delete-NT-6 × helix did not affect the total protein levels of ICAM1 and CTSS (Fig. 5g). Rather, ICAM1 and CTSS expressions were decreased in the cell supernatant medium from AQP1-delete-NT-6 × helix over-expressing cells (Fig. 5h). The supernatant from AQP1-delete-NT-6 × helix over-expressing cells reversed AQP1-induced migration and invasion abilities (*P* = 0.0145 for migration, *P* = 0.0072 for invasion, two-tailed Student’s *t* test, ^*^P < 0.05, ^**^P < 0.01, Fig. 5i, j). These results indicate that the NT-6 × helix domain of AQP1 was necessary for cell secretion of prometastatic proteins. Thus, we proposed that AQP1 induced local invasion by augmenting the cellular release of pro-metastatic proteins ICAM1 and CTSS by binding with ANXA2.

### Rab1b was shown necessary for the AQP1-induced ICAM1/CTSS secretion

**Fig. 6:**
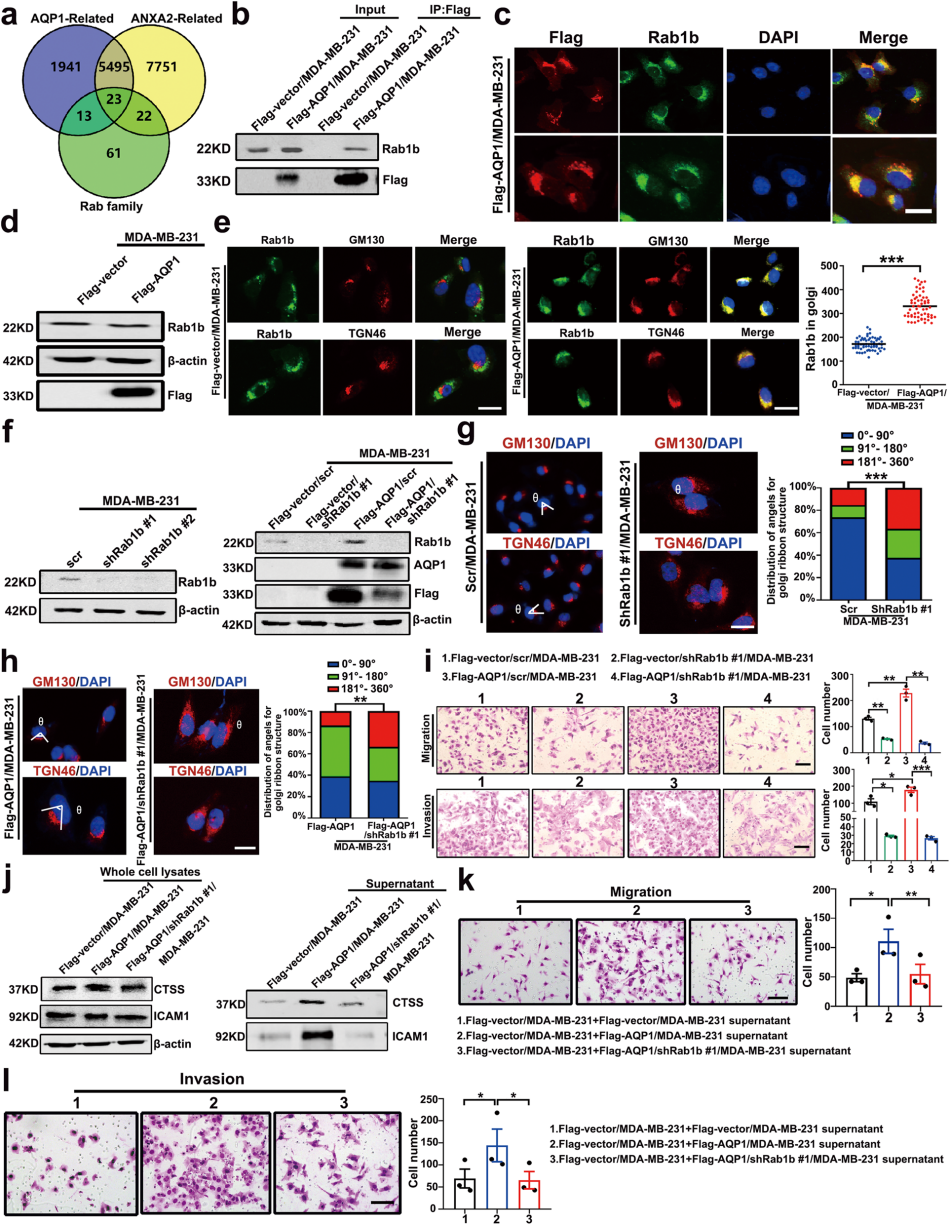
Rab1b was shown necessary for the AQP1-induced ICAM1/CTSS secretion. **a** The Venn diagram showed the intersection of three datasheets using 817 RNA-seq data from TCGA database. **b** Immunoprecipitation experiments were performed by using an anti-Flag M2 affinity gel and then immunoblotted with anti-Rab1b or anti-Flag. **c** Immunofluorescent staining of Flag together with Rab1b in Flag-AQP1/MDA-MB-231 cells. Scale bar = 20 µm. **d** Western blot analysis of Rab1b expression in Flag-vector/MDA-MB-231 and Flag-AQP1/MDA-MB-231 cells. **e** Immunofluorescent assay showed the cellular location of Rab1b in Flag-vector/MDA-MB-231 and Flag-AQP1/MDA-MB-231 cells (*n* = 60 cells per group, two-tailed Student’s *t* test, ^***^P < 0.001). Scale bar = 20 μm. **f** Western blot analysis of indicated cell clones. Scr/MDA-MB-231 and Flag-vector/scr/MDA-MB-231 cells were used as control. **g**-**h** Immunofluorescent staining of GM130/TGN46 and DAPI in 4 different cell clones. In the right panel showed the distribution of cells with different ranges of Golgi ribbon angle (Ɵ: 0°–360°), respectively. ^**^P < 0.01, ^***^P < 0.001. Scale bar = 20 μm. **i** The abilities of migration and invasion were detected using 4 indicated cell clones. Quantitative results were analyzed in the right panel. Each bar represented the mean ± SEM from three independent experiments (two-tailed Student’s *t* test, ^*^P < 0.05, ^**^P < 0.01, ^***^P < 0.001). Scale bar = 100 μm. **j** Western blot analysis of the whole cell lysates or supernatant medium samples isolated from 3 indicated cells was performed to quantify ICAM1 and CTSS proteins. **k**-**l** Representative migration **(k)** or invasion **(l)** images from Flag-vector/MDA-MB-231 cells treated with the supernatant medium 3 different cells (two-tailed Student’s *t* test, ^*^P < 0.05, ^**^P < 0.01). Each bar represented the mean ± SEM from three independent experiments. Scale bar = 100 µm. All in vitro experiments were repeated at least 3 times

To gain further secretion mechanistic insights into the cellular function of AQP1, we sought to identify protein (s) that could directly regulate the cells secretion and was localized in the Golgi apparatus. Rab can control membrane budding and formation of transport vesicles, which is essential for the localization and function of membrane and secretory proteins such as hormones, growth factors, and their membrane receptors [39, 40]. Based on the RNA sequencing data from the 817 breast cancer samples in the TCGA database, we obtained AQP1- and ANXA2-related genes. A Venn diagram showed an intersection of 23 genes among the AQP1-related group, the ANXA2-related group, and the Rab family group (Fig. 6a). In Supplementary Table S10, 23 genes were listed.

Rab1b was important for the Golgi secretion [20]. Co-immunoprecipitation experiments showed that AQP1 interacted with Rab1b (Fig. 6b). Immunofluorescence staining revealed AQP1 co-localized with Rab1b in the perinuclear Golgi region of breast cancer cells (Fig. 6c). To address the functional significance of the interaction between AQP1 and Rab1b, we examined the effect of AQP1 on the Rab1b. By western blot analysis and immunofluorescence staining, we determined the effect of the AQP1 on the cellular expression and cellular localization of Rab1b. The results showed that over-expressing AQP1 did not affect the total amount of Rab1b but increased Rab1b location to the Golgi apparatus membrane (^***^P < 0.001, Fig. 6d, e). We then generated Rab1b-specific shRNAs to silence the endogenous Rab1b expression (shRab1b) in the MDA-MB-231, Flag-vector/MDA-MB-231, and Flag-AQP1/MDA-MB-231 cells (Fig. 6f). The shRab1b #1, was adopted for further study. To further explore the effect of Rab1b on the Golgi, we performed immunofluorescence assay. The results showed that down-regulation of Rab1b expression caused punctate Golgi apparatus morphology (^***^P < 0.001, Fig. 6g), which was consistent with previous study [41]. As expected, reduction of expression of Rab1b reversed AQP1-induced Golgi extension (^**^P < 0.01, Fig. 6h). In Supplementary Fig. 7, when treated with F-actin inhibitor latrunculin B in AQP1-overexpressing cells, Golgi reversed extend phenotype. It was unexpected, however, in Rab1b knockdown cells and Rab1b knockdown AQP1-overexpressing cells, latrunculin B treatment did not reverse shRab1b-induced Golgi dispersion (Supplementary Fig. 7). Migration and invasion assays verified that Rab1b knockdown abrogated the AQP1-mediated enhancement of migration and invasion capacities (two-tailed Student’s* t* test, ^*^P < 0.05, ^**^P < 0.01, ^***^P < 0.001, Fig. 6i). These findings showed that Rab1b acted as a downstream effector of AQP1-mediated metastasis. Rab1b is a GTPase that cycles between a guanosine diphosphate (GDP)-bound inactive state bound to GDP-dissociation inhibitor alpha in the cytoplasm and a GTP-bound active state in the Golgi apparatus, where it is required for Golgi function [42]. To find out whether Rab1b knockdown affected the Golgi secretory capacity in AQP1-overexpressing cells, we performed western blot analysis, we found that downregulation of Rab1b caused decreased secretion of ICAM1 and CTSS without changing the overall intracellular levels of these two proteins (Fig. 6j). Migration and invasion assays showed that the cells treated with the supernatant medium of AQP1-overexpressing, Rab1b knockdown cells exhibited decreased cell migration and invasion capacities (two-tailed Student’s *t* test, ^*^P < 0.05, ^**^P < 0.01, Fig. 6k, l). We found that AQP1 interacted with Rab1b and recruited Rab1b to the Golgi apparatus. Rab1b was shown necessary for AQP1-induced ICAM1 and CTSS secretion.

### AQP1, ANXA2 and Rab1b formed a ternary complex

**Fig. 7:**
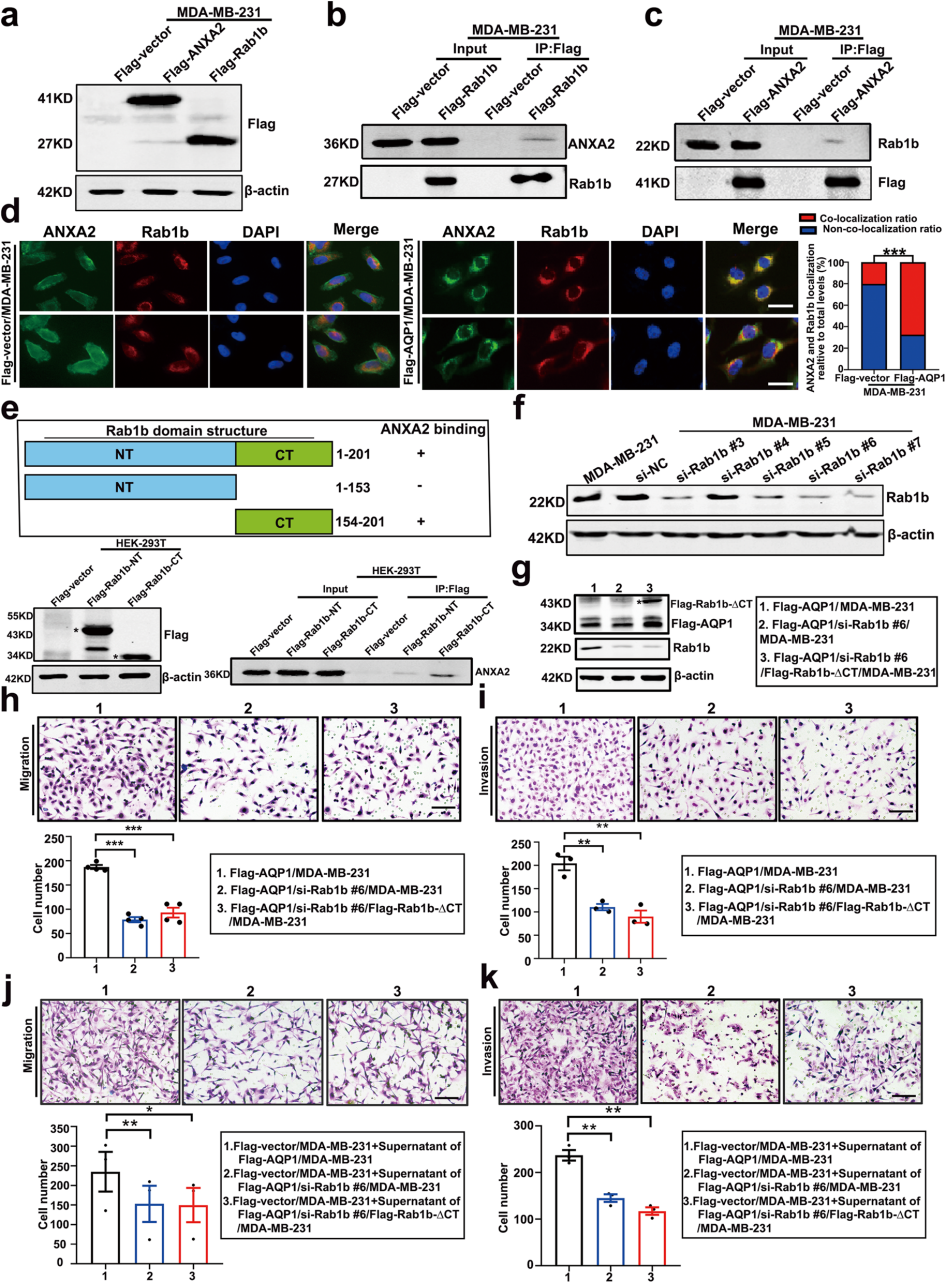
AQP1, ANXA2 and Rab1b formed a ternary complex. **a** Western blot analysis of MDA-MB-231 cells stably transfected Flag-vector, Flag-Rab1b and Flag-ANXA2 was performed using anti-flag and anti-β-actin antibodies. **b**-**c** Co-immunoprecipitation experiments confirmed the interactions between ANXA2 and Rab1b. **d** The colocalization of ANXA2 and Rab1b were detected in Flag-vector/MDA-MB-231 cells and Flag-AQP1/MDA-MB-231 cells by Immunofluorescence experiments (*n* = 166–178 cells per group, two-tailed Student’s *t* test, ^***^P < 0.001). Scale bar = 20 μm. **e** Co-immunoprecipitation experiments confirmed the interactions between ANXA2 and the different domain structure fragments of Rab1b. The Flag-Rab1b-NT/HEK-293 T and Flag-Rab1b-CT/HEK-293 T cells were also tagged with GFP. **f** Western blot was conducted to determine the expression of Rab1b in MDA-MB-231 cells transfected with si-Rab1b. NC: negative control. **g** Western blot analysis of Flag-AQP1/si-Rab1b #6/MDA-MB-231 cells transfected Flag-Rab1b-∆CT. Flag-Rab1b-∆CT/MDA-MB-231 cells were also tagged with GFP. **h**-**i** The abilities of migration and invasion were detected using Flag-AQP1/siRab1b #6/MDA-MB-231 and Flag-AQP1/siRab1b #6/Rab1b-∆CT/MDA-MB-231 cells. Each bar represented the mean ± SEM from three or four independent experiments (two-tailed Student’s *t* test, ^**^P < 0.01, ^***^P < 0.001). Scale bar = 100 μm. **j**-**k** The abilities of migration and invasion were detected using cell supernatants medium of Flag-AQP1/siRab1b #6/MDA-MB-231 and Flag-AQP1/siRab1b #6/Rab1b-∆CT/MDA-MB-231 cells. Each bar represented the mean ± SEM from three independent experiments (two-tailed Student’s *t* test, ^*^P < 0.05, ^**^P < 0.01). Scale bar = 100 μm. All in vitro experiments were repeated at least 3 or 4 times

To determine the relation between ANXA2 and Rab1b, we surveyed relevant literatures and tried to find a linkage. Mass spectrometry analysis of ANXA2-interacting proteins showed a possible interaction between ANXA2 and Rab1b [43]. We performed an immunoprecipitation assay by constructing MDA-MB-231 cell lines stably over-expressing ANXA2 or Rab1b (Fig. 7a). Co-immunoprecipitation experiments showed that ANXA2 and Rab1b interacted with each other (Fig. 7b, c). Western blot and immunofluorescence analysis showed that ANXA2 and Rab1b did not influence the expression and location of each other (Supplementary Fig. 8a-d). This was expected, because the co-localization of ANXA2 and Rab1b was increased in AQP1-overexpressing cells (Fig. 7d). In Fig. 7d, 20.22% cells showed the co-localization of ANXA2 and Rab1b in control group, but 67.47% cells in AQP1 overexpression group. Based on this, we concluded that AQP1, ANXA2, and Rab1b could form a tripartite complex in the Golgi apparatus.

To determine the region of Rab1b necessary for the interaction of ANXA2 with Rab1b, we constructed 2 deletion mutants of Rab1b and transfected them into HEK-293 T cells, and co-immunoprecipitation experiments confirmed that the C-terminal region of Rab1b interacted with ANXA2 (Fig. 7e).

We next asked if binding between ANXA2 and Rab1b is required for the pro-metastatic function of AQP1. We transfected 5 siRNAs to knockdown the expression of Rab1b in MDA-MB-231 cells (Fig. 7f). siRab1b #6 was adopted for further study. The cell lines over-expressing Rab1b-delete-CT in AQP1 over-expressing, Rab1b knockdown cells (Flag-AQP1/si-Rab1b#6/Flag-Rab1b-∆CT/MDA-MB-231) were constructed (Fig. 7g). Knocking down Rab1b in AQP1-overexpressing cells decreased cells migration and invasion abilities, while over-expressing Rab1b-delete-CT in AQP1 over-expressing, Rab1b knockdown cells did not reverse AQP1 overexpressing, Rab1b knockdown-induced migration and invasion abilities (two-tailed Student’s *t* test, ^**^P < 0.01, ^***^P < 0.001, Fig. 7h, i). These results indicated that the CT domain of Rab1b was necessary for AQP1-induced cell migration and invasion. The cell function assays using supernatant medium also confirmed the above results (two-tailed Student’s *t* test, ^*^P < 0.05, ^**^P < 0.01, Fig. 7j, k). They demonstrated that the CT domain of Rab1b was necessary for the AQP1-induced cell secretion. Our results indicated that AQP1, ANXA2, and Rab1b formed a ternary complex in the Golgi apparatus to induce breast cancer local invasion.

### Both in vivo assay and clinical analysis data confirmed that AQP1 could induce breast cancer progression by interacting with ANXA2 and Rab1b

**Fig. 8:**
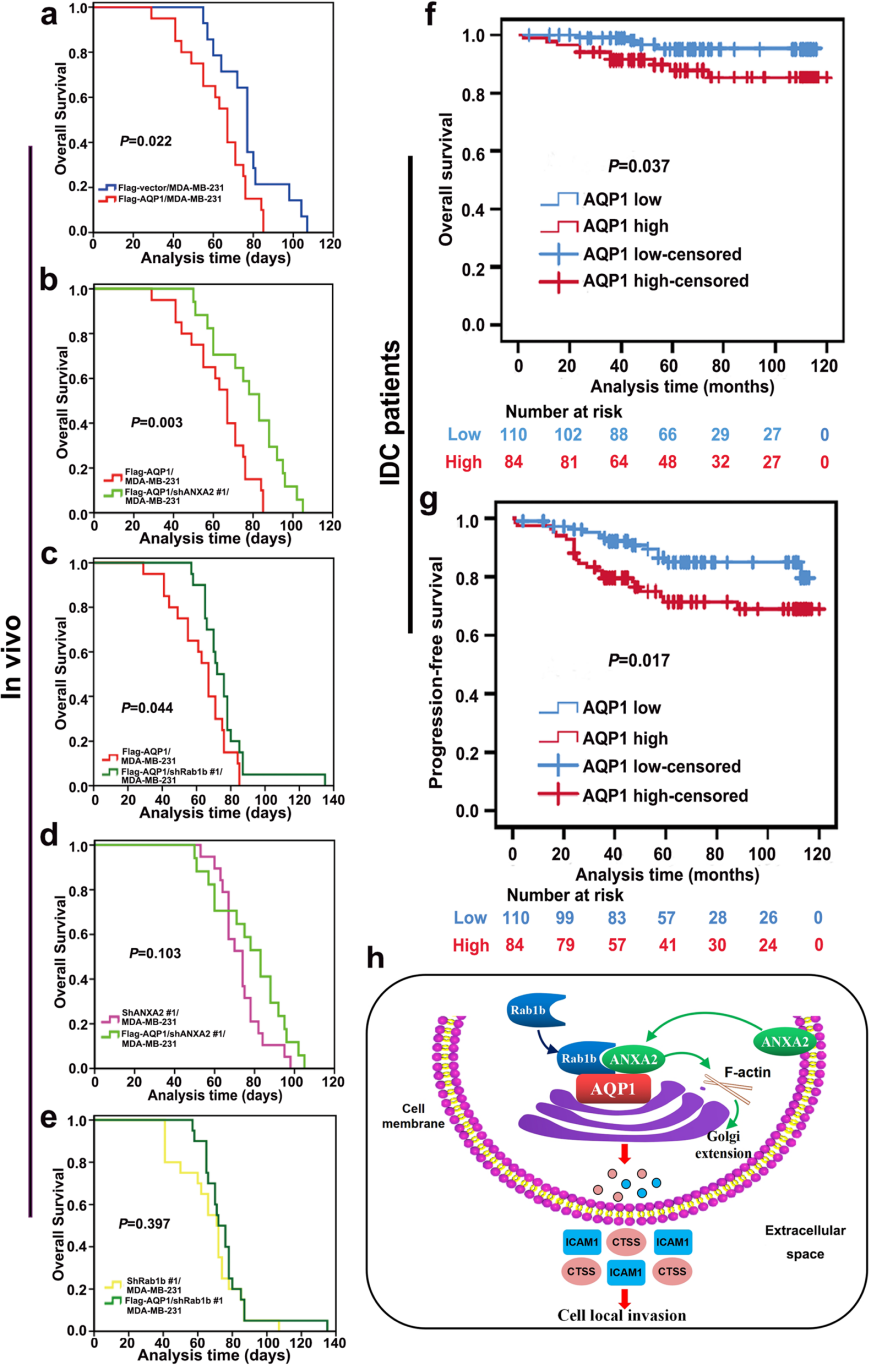
Both in vivo assay and clinical analysis data confirmed that AQP1 induced breast cancer progression by interacting with ANXA2 and Rab1b. **a**-**e** Survival times between animal groups were compared (log-rank test). The Flag-AQP1/MDA-MB-231 (*n* = 20) mice group had a shorter survival time compared with Flag-vector/MDA-MB-231 (*n* = 14) mice group **(a)**; Flag-AQP1/shANXA2 #1/MDA-MB-231 (*n* = 17) mice group **(b)** and Flag-AQP1/shRab1b #1/MDA-MB-231 (*n* = 20) mice group **(c)** also had a longer survival time when compared with Flag-AQP1/MDA-MB-231 (*n* = 20) mice group; The survival time was not changed between shANXA2 #1/MDA-MB-231 (*n* = 19) and Flag-AQP1/shANXA2 #1/MDA-MB-231 (*n* = 17) mice group **(d)**; Comparison of survival curve between shRab1b #1/MDA-MB-231 (*n* = 20) and Flag-AQP1/shRab1b #1/MDA-MB-231 (*n* = 20) mice group **(e)**. **f**, **g** Kaplan–Meier analysis of survival of 194 IDC patients with AQP1 high cytoplasmic expression or AQP1 low cytoplasmic expression (log-rank test). **h** Overview of the AQP1/ANXA2/Rab1b signaling pathway in breast cancer local invasion

To further investigate the effect of up-regulated AQP1 on the animal survival, we performed experiments using a xenograft nude mouse model. The terminal point of this experiment was the end of each animal's survival. In details, four-week-old female nude mice were randomly divided into 6 groups. 6 kinds of xenograft tumors (which were described in materials and methods) were cut into small pieces (2 × 2 mm) and subsequently anchored to the third pair of fat pads on the right side of new nude mice. The survival time of animals among different groups was compared. We found that Flag-AQP1/MDA-MB-231 mice group (*n* = 20) had a shorter survival time than the control group (*P* = 0.022, *n* = 14) (Fig. 8a). Flag-AQP1/shANXA2 #1/MDA-MB-231 (*n* = 17) or Flag-AQP1/shRab1b #1/MDA-MB-231 (*n* = 20) mice group had increased survival time compared with Flag-AQP1/MDA-MB-231 mice group (*P* = 0.003, *P* = 0.044, Fig. 8b, c). But survival time was unchanged in Flag-AQP1/shANXA2 #1/MDA-MB-231 mice group (*n* = 17) compared with shANXA2 #1/MDA-MB-231 (*n* = 19) mice group (*P* = 0.103, Fig. 8d). Similar results were also observed in Flag-AQP1/shRab1b #1/MDA-MB-231 mice group (*n* = 20) compared with shRab1b #1/MDA-MB-231 (*n* = 20) mice group (*P* = 0.397, Fig. 8e).

Breast cancer patients with high expression of AQP1 had worse survival than patients with low AQP1 expression, as shown in Kaplan–Meier survival curves in GEPIA (http://gepia.cancer-pku.cn) (*P* = 0.024, Supplementary Fig. 9a). Kaplan–Meier analyses from the Kmplot database revealed that high expression levels of ANXA2, Rab1b, CTSS, and ICAM1 were also associated with poor survival prognosis (log-rank test, Supplementary Fig. 9b-e). Correlation analysis using 194 IDC patients or the GEPIA database indicated that AQP1 was positively correlated with ANXA2, Rab1b, CTSS, and ICAM1 in breast cancer patients (Supplementary Fig. 10a-d and Supplementary Table S8). In order to explore the role of AQP1 in breast cancer prognosis, we assessed the overall survival time of the 194 IDC patients with complete clinical follow-up. Patients with high levels of AQP1 showed shorter overall survival (OS) (*P* = 0.037, Fig. 8f) and progression-free survival (PFS) (*P* = 0.017, Fig. 8g). Both in vivo assay and clinical analysis data confirmed that AQP1 could induce breast cancer progression by interacting with ANXA2 and Rab1b.

## Discussion

As a transmembrane ion channel, the main function of AQP1 is to transfer water, glycerol and other small solutes across cellular membranes [44, 45]. Our previous study revealed that water channel protein AQP1 was mainly localized in the cytosol of breast cancer cells and promoted breast cancer progression [6]. Yamazato et al. also confirmed our finding in esophageal squamous cell carcinoma [46]. Furthermore, our present study discovered that AQP1 with ANXA2 and Rab1b formed a ternary complex in Golgi apparatus to induce breast cancer progression. Similarly, TRP vanilloid-1 (TRPV1), a calcium ion channel protein on the cell membrane, serving to non-selectively permeate calcium ion from the extracellular region to the cytoplasm and also was found to localize in glioma cells cytoplasm to activate mitochondrial-dependent apoptosis of glioma cells [47]. These indicated that some certain ion channel proteins not only exerted their physiological functions in cell membrane, but also played a critical role in tumor progression.

As previously established, small molecules targeting AQP1 have been investigated. Xiang Y, et al. reported that Acetazolamide could inhibit the expression AQP1 protein and angiogenesis [48]. Subsequent studies demonstrated that Ginsenoside Rg3 attenuated cell migration via inhibition of AQP1 expression in PC-3M prostate cancer cells [49]. Furthermore, AqB007 and AqB011 could selectively block the AQP1 ion channel conductance and slow cancer cell migration [50]. Besides, a study reported that synthetic small molecules AqB013 and AqB050 exhibited an anti-tubulogenic effect through inhibition of AQP1-mediated cell migration and induction of apoptosis [51]. Such AQP1 inhibitors probably have potential effects on tumor progression.

Several studies indicated that AQP1 could be involved in cellular signaling. AQP1 could facilitate proliferation and invasion of gastric cancer cells via GRB7-mediated ERK and Ras activation [52]. AQP1 expression induced re-localization of actin protein and activation of RhoA and Rac, meanwhile AQP1 could down-regulate the expression of hypoxia-related genes [53, 54]. Besides, AQP1 could interact with Lin-7/β-catenin, affecting the organization of cytoskeleton to promote cell migration [55]. Our study report here suggested a new molecular mechanism of AQP1 that promoted breast cancer local invasion.

In our study, no endogenous AQP1 expression was detected in MDA-MB-231 and T47D cell lines by Western blot. This loss of AQP1 in long-term tumor cell cultures and cell lines is likely a result of culture condition. It has been shown that AQP1 expression is regulated via osmotic response elements and hypertonicity. AQP1 was downregulated in cells which were not stimulated by constant changes in osmolarity, while AQP1 was upregulated by a hypertonic challenge in cells [56–59]. Besides, since endogenous AQP1 was undetectable in MDA-MB-231 and T47D cell lines, we further utilized two kinds of primary breast cancer cells to confirm the localization and function of AQP1 (Fig. 1f-h). The results showed that the localization and function of exogenous AQP1 is consistent with that of endogenous AQP1.

We observed that AQP1 could localize at the Golgi apparatus. But how AQP1 localized at the Golgi needs to be explored for future research. It is known that the translocation of AQP1 to the plasma membrane depends on its phosphorylation status [60]. The phosphorylation levels of AQP1 in breast cancer cells exhibiting a high cytoplasmic pool were likely lower as compared to cells expressing mainly a plasma membrane pool of AQP1.

Rab1b is the founding member of the Rab small GTPase family, the conversion of Rab1b between the inactive GDP-bound and active GTP-bound forms regulates localization of Rab1b proteins to either cytosol or membranes [61]. Furthermore, our results also showed that the abundance of Rab1b in the Golgi apparatus was increased in AQP1 over-expressing cells, which probably due to the consequence of enhanced GTP-bound Rab1b.

ANXA2 is a calcium-dependent phospholipid binding protein, it could directly interact with actin and involve in maintain the dynamic actin cytoskeleton [37]. ANXA2 could inhibit actin depolymerisation, and incubation of growing F-actin filaments with ANXA2 resulted in the formation of shorter filaments. Besides, Ozorowski et al. reported that the C terminus of ANXA2 mediated binding to F-actin [62]. In the present study, we observed the extended phenotype of Golgi phenotype was reversed when the AQP1-overexpressing cells were treated with the F-actin inhibitor latrunculin B. Similar effects were also observed in ANXA2-knockdown AQP1-overexpressing cell (Fig. 4d). Therefore, AQP1-induced Golgi apparatus extension was ANXA2/F-actin dependent. Actin filaments are involved in the maintenance of Golgi apparatus morphology [35, 36] Depolymerization of actin could alter Golgi morphology have been reported in series of studies [36, 63]. Therefore, we speculate AQP1 combined with ANXA2 to regulate Golgi apparatus morphology through regulating actin rearrangement.

AQP1, as a water channel protein, is normally localized on the cell membrane. However, in breast cancer cells, AQP1 is mainly localized in the cellular cytoplasm. According to the result of Fig. 3k, we found ANXA2 mainly located in cells membrane in AQP1 deficient cells, while it translocated to cytoplasm when AQP1 was overexpressed in cytoplasm. Therefore, we speculated that AQP1 probably bound with ANXA2 and carried ANXA2 to the cytoplasm. Meanwhile, according to the result of Fig. 6e, we found Rab1b rarely located on Golgi apparatus in AQP1 deficient cells, while it translocated to Golgi apparatus when AQP1 was overexpressed in cytoplasm. Therefore, we speculate that AQP1 probably bound with Rab1b and carried it from cytoplasm to Golgi apparatus. Based on the above results, it showed that AQP1 could recruit ANXA2 and Rab1b to Golgi apparatus and formed a ternary complex.

Intercellular adhesion molecule 1 (ICAM1) is a cell surface glycoprotein. Rosette et al. reported that ICAM1 exerted the function of promoting breast cancer cells invasion. Cells incubation with an antibody against ICAM1 inhibited the invasion abilities of the highly metastatic MDA-MB-435 cell line in a dose-dependent manner [64]. In triple negative breast cancer (TNBC), the ICAM1 antibody significantly reduced cell migration and ICAM1 could act as a molecular target for TNBC [23]. A recent study reported that ICAM1 bound with integrins to activate the epithelial-to-mesenchymal transition process through TGF-β/SMAD signaling, ultimately enhancing breast cancer cell invasion [65]. Consistent with previous studies, our study found that ICAM1 could promote breast cancer cells local invasion.

The cysteine protease cathepsin S (CTSS) is one of a family of 11 cysteine cathepsin proteases. Previous studies have demonstrated the key role of CTSS in cancer cell invasion. Sevenich et al. found that CTSS could act as a regulator of breast-to-brain metastasis and CTSS inhibition reduces brain metastasis formation in breast cancer [28]. In TNBC, inhibition of CTSS and MMP-9 suppresses cells invasion and metastasis [25]. Besides, up-regulated levels of CTSS in TNBC tissues have been demonstrated to play a critical role in accelerating tumor invasion [66]. In line with previous studies, our study demonstrated that down-regulated CTSS inhibited breast cancer cells local invasion.

Our present study reported that AQP1 could promote Golgi apparatus extension, leading to increased cell secretion of ICAM1 and CTSS and increasing breast cancer cell migration and invasion. Halberg et al. also found that highly metastatic cells exhibited a more extended Golgi structure compared with their poorly metastatic parental cell populations [20]. Such morphological and functional changes of Golgi apparatus have been proved to promote metastasis capacity of cancer cells [19].

## Conclusion

In conclusion, this study demonstrates that AQP1 is critical to the local invasion in breast cancer metastasis (Fig. 8h). Therefore, targeting AQP1 offers promises in breast cancer treatment.

## Supplementary Information

**Additional file 1: Supplementary Fig. 1.** Cytoplasmic expression of AQP1 was positively correlated with breast cancer progression. (a) Patients who had a recurrence or metastasis had a higher AQP1 cytoplasmic expression (62.2% vs 38.9%, *P* = 0.009). Cyto-AQP1: cytoplasmic AQP1 expression. (b) Patients who had a lymph node metastasis (*n* > 4) had a higher AQP1 cytoplasmic expression (50.0% vs 38.1%, *P* = 0.038). Cyto-AQP1: cytoplasmic AQP1 expression. (c-d) The relationship between pT stage and AQP1 cytoplasmic expression. Cyto-AQP1: cytoplasmic AQP1 expression. (e) The tumor volume in Flag-vector/MDA-MB-231 and Flag-AQP1/MDA-MB-231 mice group. Values were expressed as mean ± SD (two-tailed Student’s *t* test and two-way ANOVA, ^**^P < 0.01, ^***^P < 0.001). (f) Quantitation of the percentage of Ki67-positive cells in tumor sections of Flag-vector/MDA-MB-231 and Flag-AQP1/MDA-MB-231 mice group. Two-tailed Student’s *t* test, ^*^P < 0.05. **Supplementary Fig. 2. **Down-regulated expression of AQP1 decreased breast cancer migration and invasion abilities in AQP1-overexpressing MDA-MB-231 cells. (a) Western blot analysis of the expression of AQP1 in Flag-AQP1/MDA-MB-231 cells transfected with AQP1 shRNA. GAPDH was the loading control. (b-c) The abilities of migration and invasion were detected using Flag-AQP1/MDA-MB-231 and Flag-AQP1/shAQP1/MDA-MB-231 cells. Values were expressed as mean ± SEM from three independent experiments (two-tailed Student’s *t* test, ^**^P<0.01). Scale bar = 100 μm. (d-e) Migration and invasion assay showed that Flag-vector/MDA-MB-231 cells treated with the supernatant of Flag-AQP1/shAQP1/MDA-MB-231 cells reversed the promoted phenotype compared with Flag-AQP1/MDA-MB-231 cells (two-tailed Student’s *t* test, ^*^P<0.05, ^***^P<0.001). Each bar represented the mean ± SEM from three independent experiments. Scale bar=100 μm. **Supplementary Fig. 3. **Over-expression AQP1 increased breast cancer invasion abilities in T47D breast cancer cells. (a) Western blot analysis of the expression of AQP1 in Flag-vector/T47D cells and Flag-AQP1/T47D cells. β-actin was the loading control. (b) The abilities of invasion were detected using Flag-vector/T47D and Flag-AQP1/T47D cells. Values were expressed as mean ± SEM from seven independent experiments (two-tailed Student’s *t* test, ^**^P<0.01). Scale bar=100 μm. (c) Invasion assay showed that Flag-vector/T47D cells treated with the supernatant of Flag-AQP1/T47D cells exhibited promoted phenotype compared with Flag-AQP1/T47D cells (two-tailed Student’s *t* test, ^*^P<0.05). Each bar represented the mean ± SEM from four independent experiments. Scale bar=100 μm. (d) Immunofluorescent assay showed the colocation of AQP1 and ANXA2 in Flag-AQP1/T47D cells. Scale bar=20 μm. All experiments were independently repeated at least for three times. **Supplementary Fig. 4.** Gene enrichment analysis of AQP1 related genes. (a) 1805 differentially expression genes related to AQP1 were associated with extracellular matrix and secretion in up_keywords category of DAVID tool. (b) The biological process enrichment analysis of 1805 differentially expressed genes. (c) The molecular function enrichment analysis of AQP1 related genes. (d) The cellular component enrichment analysis of AQP1 related genes. (e) The KEGG pathway enrichment analysis of AQP1 related genes. **Supplementary Fig. 5.** Gene enrichment analysis of 76 proteins in the supernatant of AQP1-overexpressing MDA-MB-231 cells. (a) Representative TEM images of Golgi structure of Flag-vector/MDA-MB-231 and Flag-AQP1/MDA-MB-231 cells. Scale bar = 500 nm. (b) Mass spectrometry analysis showed the differentially expressed proteins between the supernatant of Flag-vector/MDA-MB-231 cells and Flag-AQP1/MDA-MB-231 cells. (c) The biological process enrichment analysis of 76 differentially expressed genes. (d) The molecular function enrichment analysis of 76 differentially expressed genes. (e) GO cellular component enrichment results of 76 proteins only expressed in the supernatant of Flag-AQP1/MDA-MB-231 cells. (f) Heatmap of the analyzed differentially expressed proteins using 15 AQP1 high expression cases and 15 AQP1 low expression cases in 817 RNA-seq data. **Supplementary Fig. 6. **Over-expression ANXA2 exhibited extend Golgi apparatus. (a) Western blot analysis of Hela cells transfected with Flag-AQP1. (b) Western blot analysis of GFP-AQP1/MDA-MB-231 cells transfected with Flag-ANXA2. Flag-vector/MDA-MB-231 cells were regarded as negative control. (c) Western blot analysis of MDA-MB-231 cells transfected with indicated ANXA2 shRNAs or control shRNA (scr). (d) Western blot analysis of indicated cell clones. Flag-vector/scr/MDA-MB-231 cells were used as a control. (e) Western blot analysis of MDA-MB-231 cells stably transfected with Flag-ANXA2. Flag-vector/MDA-MB-231 cells were regarded as negative control. (f) Immunofluorescent staining of GM130/TGN46 and DAPI in Flag-vector/MDA-MB-231 and Flag-AQP1/MDA-MB-23 cells. Quantification of the distribution of cells with different ranges of Golgi ribbon angle (Ɵ: 0°–360°) (*n* = 77-86 cells per group, ^***^P < 0.001). Scale bar = 20 μm. All experiments were independently repeated for three times. **Supplementary Fig. 7. **The extend Golgi morphology induced by AQP1 was not through Rab1b-F-actin axis. Immunofluorescence analysis showed that the Golgi morphology was not altered in shRab1b #1/MDA-MB-231 and Flag-AQP1/shRab1b #1/MDA-MB-231 cells when treated with F-actin depolymerization inhibitor latrunculin B, compared with the cells treated with DMSO. Scale bar=20 μm. **Supplementary Fig. 8.** Both expression and location of ANXA2 and Rab1b independently of each other. (a-b) Western blot assays were performed to detect Rab1b or ANXA2 expression in different cell clones. (c-d) Immunofluorescence experiments were performed to analyze the location of Rab1b or ANXA2 in different cell clones (*n*>60 cells per group, two-tailed Student’s *t* test). Scale bar=20 μm. All experiments were independently repeated for three times. **Supplementary Fig. 9. **The effect of the expression of AQP1/ANXA2/Rab1b/CTSS/ICAM1 on the prognosis of breast cancer patients. (a) Kaplan–Meier analysis showed the association between AQP1 expression and overall survival of breast cancer patients in GEPIA database (log-rank test). (b) Kaplan–Meier analysis showed the association between ANXA2 expression and overall survival of breast cancer patients in Kaplan-Meier plotter database (log-rank test). (c) Kaplan–Meier analysis showed the association between Rab1b expression and overall survival of breast cancer patients in Kaplan-Meier plotter database (log-rank test). (d) Kaplan–Meier analysis showed the association between CTSS expression and overall survival of breast cancer patients in Kaplan-Meier plotter database (log-rank test). (e) Kaplan–Meier analysis showed the association between ICAM1 expression and overall survival of breast cancer patients in Kaplan-Meier plotter database (log-rank test). **Supplementary Fig. 10.** Relationship of AQP1 with ANXA2, Rab1b, CTSS and ICAM1. (a) In 194 IDC patients, AQP1 had a positive correlation with ANXA2. (b-d) Correlation analysis using GEPIA (http://gepia.cancer-pku.cn) indicated that AQP1 was positively correlated with Rab1b, CTSS and ICAM1 in breast cancer patients.

**Additional file 2: Supplementary Table S1.** List of plasmids and RNA interference sequences used in this study. **Supplementary Table S2.** List of antibodies used in this study. **Supplementary Table S3.** Cytoplasmic AQP1 expression in IDC patients. **Supplementary Table S4.** Relationship between AQP1 cytoplasmic expression and pathological tumor size (pT). **Supplementary Table S5. **Univariate and multivariate analysis for overall survival (OS) and progression-free survival (PFS). **Supplementary Table S6.** List of proteins in the supernant of Flag-vector/MDA-MB-231 cells by mass spectrometry. **Supplementary Table S7.** List of proteins in the supernant of Flag-AQP1/MDA-MB-231 cells by mass spectrometry. **Supplementary Table S8. **Correlations among the expression of AQP1, ANXA2 and CTSS in 194 IDC patients. **Supplementary Table S9. **Relationship between AQP1 cytoplasmic expression and ANXA2 membrane expression in IDC patients (*n*=194). **Supplementary Table S10.** List of 23 genes of Rab family associated with AQP1 and ANXA2 by analysis of TCGA database.

## Data Availability

Mass spectrometry data in this study is included in this manuscript (Supplementary Tables S6, 7). All public datasets analyzed in this study are listed in the Materials and methods “Bioinformatics analysis” section.

## Abbreviations

BP: Biological process
CER: Cytosol extraction reagent
DAPI: 4’,6- Diamidino-2-phenylindole
DEG: Differentially expression genes
ECM: Extracellular matrix
FBS: Fetal bovine serum
GDP: Guanosine diphosphate
GEO: Gene Expression Omnibus
GO: Gene ontology
IDC: Invasive ductal carcinoma
IHC: Immunohistochemistry
IP: Immunoprecipitation
KEGG: Kyoto Encyclopedia of Genes and Genomes
MF: Molecular function
OS: Overall survival
PFS: Progression-free survival
STR: Short tandem repeat
TEM: Transmission electron microscopy
TNBC: Triple negative breast cancer
