# Supplementary figures and images for: Water channel protein AQP1 in cytoplasm is a critical factor in breast cancer local invasion
Source: J Exp Clin Cancer Res. 2023 Feb 20;42:49. doi: 10.1186/s13046-023-02616-1 (PMC9940370; doi:10.1186/s13046-023-02616-1)

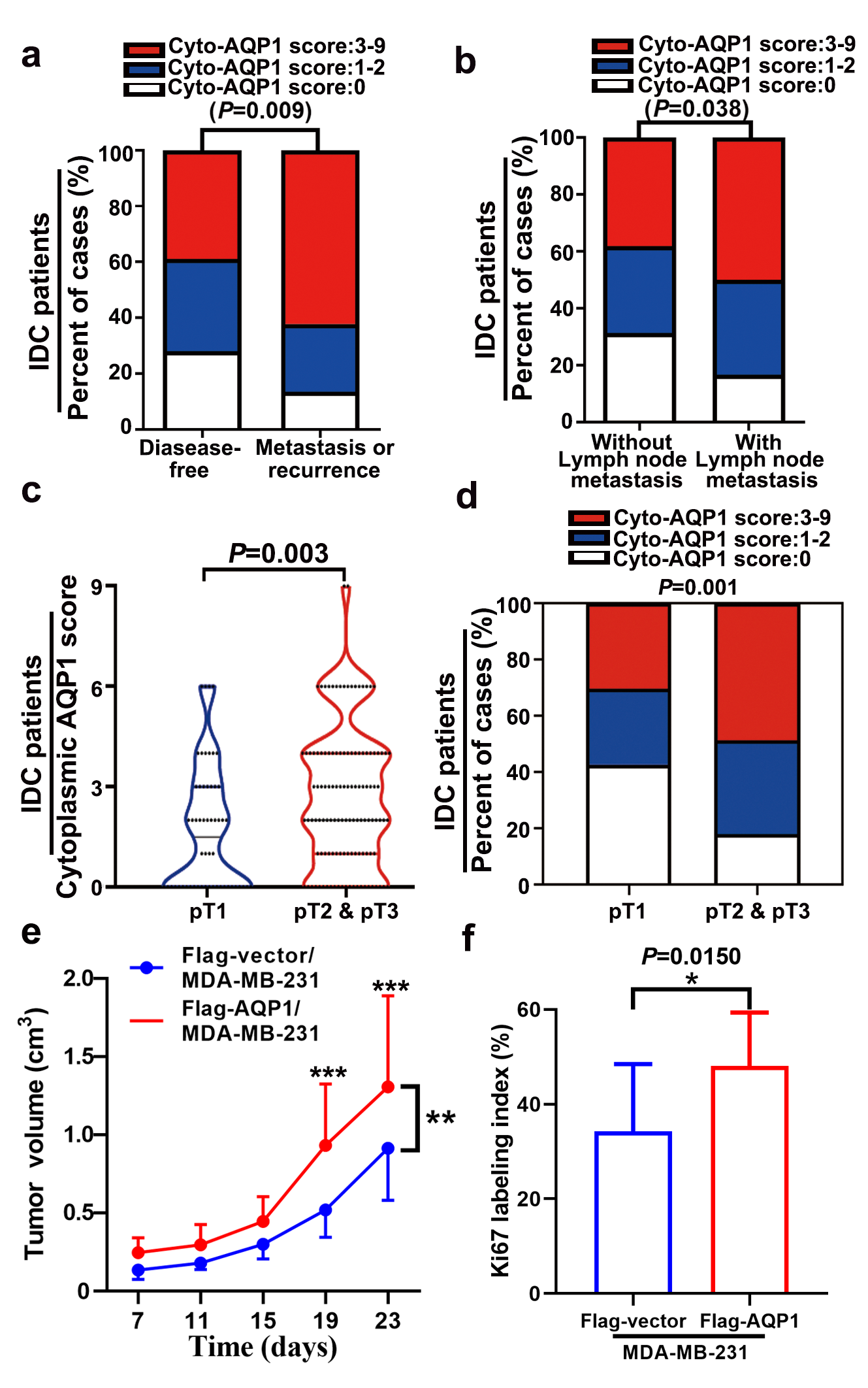

Supplement: Supplementary file 1 — Additional file 1: Supplementary Fig. 1. Cytoplasmic expression of AQP1 was positively correlated with breast cancer progression. (a) Patients who had a recurrence or metastasis had a higher AQP1 cytoplasmic expression (62.2% vs 38.9%, P = 0.009). Cyto-AQP1: cytoplasmic AQP1 expression. (b) Patients who had a lymph node metastasis (n > 4) had a higher AQP1 cytoplasmic expression (50.0% vs 38.1%, P = 0.038). Cyto-AQP1: cytoplasmic AQP1 expression. (c-d) The relationship between pT stage and AQP1 cytoplasmic expression. Cyto-AQP1: cytoplasmic AQP1 expression. (e) The tumor volume in Flag-vector/MDA-MB-231 and Flag-AQP1/MDA-MB-231 mice group. Values were expressed as mean ± SD (two-tailed Student’s t test and two-way ANOVA, **P < 0.01, ***P < 0.001). (f) Quantitation of the percentage of Ki67-positive cells in tumor sections of Flag-vector/MDA-MB-231 and Flag-AQP1/MDA-MB-231 mice group. Two-tailed Student’s t test, *P < 0.05. Supplementary Fig. 2. Down-regulated expression of AQP1 decreased breast cancer migration and invasion abilities in AQP1-overexpressing MDA-MB-231 cells. (a) Western blot analysis of the expression of AQP1 in Flag-AQP1/MDA-MB-231 cells transfected with AQP1 shRNA. GAPDH was the loading control. (b-c) The abilities of migration and invasion were detected using Flag-AQP1/MDA-MB-231 and Flag-AQP1/shAQP1/MDA-MB-231 cells. Values were expressed as mean ± SEM from three independent experiments (two-tailed Student’s t test, **P<0.01). Scale bar = 100 μm. (d-e) Migration and invasion assay showed that Flag-vector/MDA-MB-231 cells treated with the supernatant of Flag-AQP1/shAQP1/MDA-MB-231 cells reversed the promoted phenotype compared with Flag-AQP1/MDA-MB-231 cells (two-tailed Student’s t test, *P<0.05, ***P<0.001). Each bar represented the mean ± SEM from three independent experiments. Scale bar=100 μm. Supplementary Fig. 3. Over-expression AQP1 increased breast cancer invasion abilities in T47D breast cancer cells. (a) Western blot analysis of the expr [file 13046_2023_2616_MOESM1_ESM.zip › Supplementary Fig. 1.tif]

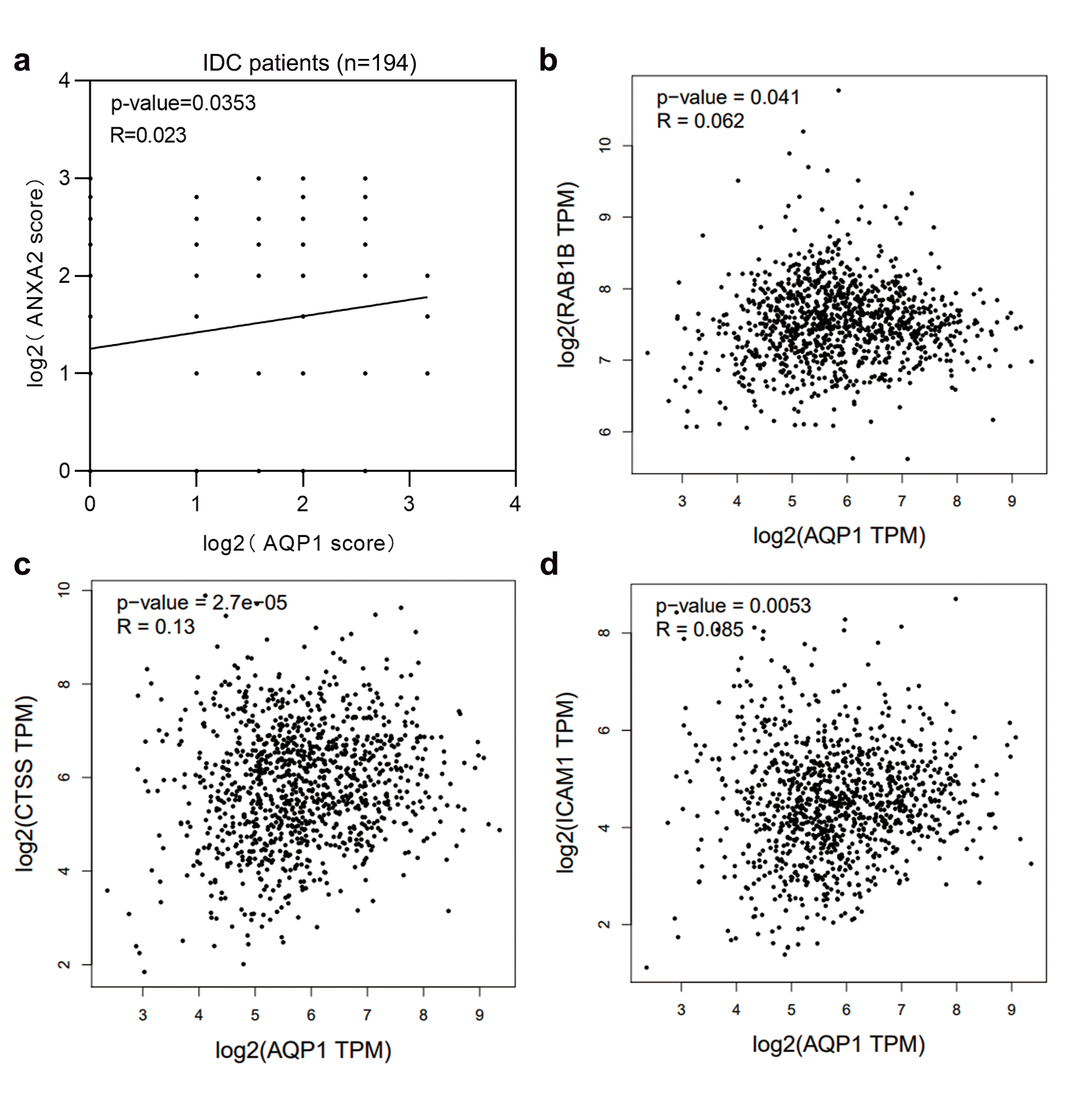

Supplement: Supplementary file 1 — Additional file 1: Supplementary Fig. 1. Cytoplasmic expression of AQP1 was positively correlated with breast cancer progression. (a) Patients who had a recurrence or metastasis had a higher AQP1 cytoplasmic expression (62.2% vs 38.9%, P = 0.009). Cyto-AQP1: cytoplasmic AQP1 expression. (b) Patients who had a lymph node metastasis (n > 4) had a higher AQP1 cytoplasmic expression (50.0% vs 38.1%, P = 0.038). Cyto-AQP1: cytoplasmic AQP1 expression. (c-d) The relationship between pT stage and AQP1 cytoplasmic expression. Cyto-AQP1: cytoplasmic AQP1 expression. (e) The tumor volume in Flag-vector/MDA-MB-231 and Flag-AQP1/MDA-MB-231 mice group. Values were expressed as mean ± SD (two-tailed Student’s t test and two-way ANOVA, **P < 0.01, ***P < 0.001). (f) Quantitation of the percentage of Ki67-positive cells in tumor sections of Flag-vector/MDA-MB-231 and Flag-AQP1/MDA-MB-231 mice group. Two-tailed Student’s t test, *P < 0.05. Supplementary Fig. 2. Down-regulated expression of AQP1 decreased breast cancer migration and invasion abilities in AQP1-overexpressing MDA-MB-231 cells. (a) Western blot analysis of the expression of AQP1 in Flag-AQP1/MDA-MB-231 cells transfected with AQP1 shRNA. GAPDH was the loading control. (b-c) The abilities of migration and invasion were detected using Flag-AQP1/MDA-MB-231 and Flag-AQP1/shAQP1/MDA-MB-231 cells. Values were expressed as mean ± SEM from three independent experiments (two-tailed Student’s t test, **P<0.01). Scale bar = 100 μm. (d-e) Migration and invasion assay showed that Flag-vector/MDA-MB-231 cells treated with the supernatant of Flag-AQP1/shAQP1/MDA-MB-231 cells reversed the promoted phenotype compared with Flag-AQP1/MDA-MB-231 cells (two-tailed Student’s t test, *P<0.05, ***P<0.001). Each bar represented the mean ± SEM from three independent experiments. Scale bar=100 μm. Supplementary Fig. 3. Over-expression AQP1 increased breast cancer invasion abilities in T47D breast cancer cells. (a) Western blot analysis of the expr [file 13046_2023_2616_MOESM1_ESM.zip › Supplementary Fig. 10.tif]

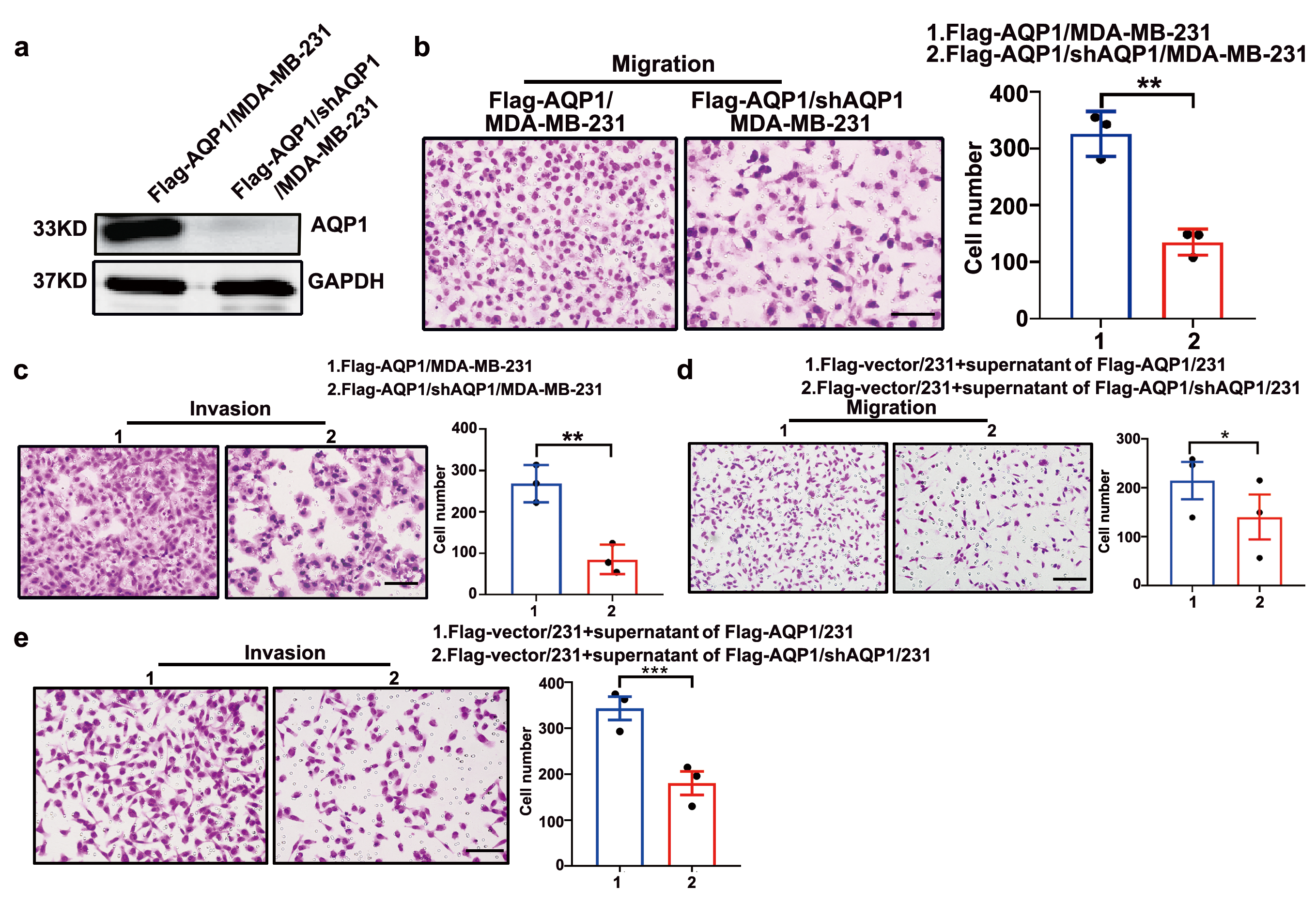

Supplement: Supplementary file 1 — Additional file 1: Supplementary Fig. 1. Cytoplasmic expression of AQP1 was positively correlated with breast cancer progression. (a) Patients who had a recurrence or metastasis had a higher AQP1 cytoplasmic expression (62.2% vs 38.9%, P = 0.009). Cyto-AQP1: cytoplasmic AQP1 expression. (b) Patients who had a lymph node metastasis (n > 4) had a higher AQP1 cytoplasmic expression (50.0% vs 38.1%, P = 0.038). Cyto-AQP1: cytoplasmic AQP1 expression. (c-d) The relationship between pT stage and AQP1 cytoplasmic expression. Cyto-AQP1: cytoplasmic AQP1 expression. (e) The tumor volume in Flag-vector/MDA-MB-231 and Flag-AQP1/MDA-MB-231 mice group. Values were expressed as mean ± SD (two-tailed Student’s t test and two-way ANOVA, **P < 0.01, ***P < 0.001). (f) Quantitation of the percentage of Ki67-positive cells in tumor sections of Flag-vector/MDA-MB-231 and Flag-AQP1/MDA-MB-231 mice group. Two-tailed Student’s t test, *P < 0.05. Supplementary Fig. 2. Down-regulated expression of AQP1 decreased breast cancer migration and invasion abilities in AQP1-overexpressing MDA-MB-231 cells. (a) Western blot analysis of the expression of AQP1 in Flag-AQP1/MDA-MB-231 cells transfected with AQP1 shRNA. GAPDH was the loading control. (b-c) The abilities of migration and invasion were detected using Flag-AQP1/MDA-MB-231 and Flag-AQP1/shAQP1/MDA-MB-231 cells. Values were expressed as mean ± SEM from three independent experiments (two-tailed Student’s t test, **P<0.01). Scale bar = 100 μm. (d-e) Migration and invasion assay showed that Flag-vector/MDA-MB-231 cells treated with the supernatant of Flag-AQP1/shAQP1/MDA-MB-231 cells reversed the promoted phenotype compared with Flag-AQP1/MDA-MB-231 cells (two-tailed Student’s t test, *P<0.05, ***P<0.001). Each bar represented the mean ± SEM from three independent experiments. Scale bar=100 μm. Supplementary Fig. 3. Over-expression AQP1 increased breast cancer invasion abilities in T47D breast cancer cells. (a) Western blot analysis of the expr [file 13046_2023_2616_MOESM1_ESM.zip › Supplementary Fig. 2.tif]

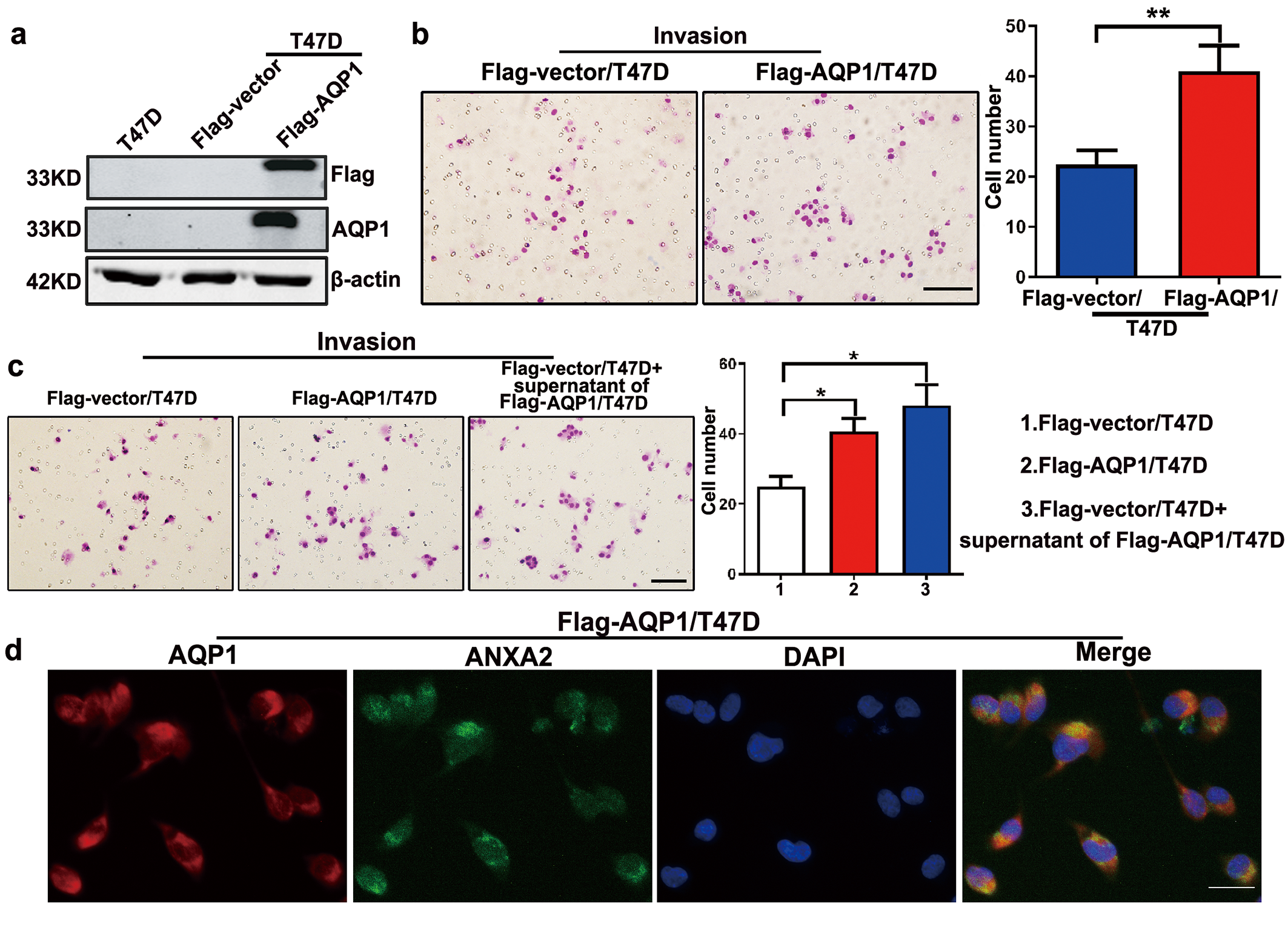

Supplement: Supplementary file 1 — Additional file 1: Supplementary Fig. 1. Cytoplasmic expression of AQP1 was positively correlated with breast cancer progression. (a) Patients who had a recurrence or metastasis had a higher AQP1 cytoplasmic expression (62.2% vs 38.9%, P = 0.009). Cyto-AQP1: cytoplasmic AQP1 expression. (b) Patients who had a lymph node metastasis (n > 4) had a higher AQP1 cytoplasmic expression (50.0% vs 38.1%, P = 0.038). Cyto-AQP1: cytoplasmic AQP1 expression. (c-d) The relationship between pT stage and AQP1 cytoplasmic expression. Cyto-AQP1: cytoplasmic AQP1 expression. (e) The tumor volume in Flag-vector/MDA-MB-231 and Flag-AQP1/MDA-MB-231 mice group. Values were expressed as mean ± SD (two-tailed Student’s t test and two-way ANOVA, **P < 0.01, ***P < 0.001). (f) Quantitation of the percentage of Ki67-positive cells in tumor sections of Flag-vector/MDA-MB-231 and Flag-AQP1/MDA-MB-231 mice group. Two-tailed Student’s t test, *P < 0.05. Supplementary Fig. 2. Down-regulated expression of AQP1 decreased breast cancer migration and invasion abilities in AQP1-overexpressing MDA-MB-231 cells. (a) Western blot analysis of the expression of AQP1 in Flag-AQP1/MDA-MB-231 cells transfected with AQP1 shRNA. GAPDH was the loading control. (b-c) The abilities of migration and invasion were detected using Flag-AQP1/MDA-MB-231 and Flag-AQP1/shAQP1/MDA-MB-231 cells. Values were expressed as mean ± SEM from three independent experiments (two-tailed Student’s t test, **P<0.01). Scale bar = 100 μm. (d-e) Migration and invasion assay showed that Flag-vector/MDA-MB-231 cells treated with the supernatant of Flag-AQP1/shAQP1/MDA-MB-231 cells reversed the promoted phenotype compared with Flag-AQP1/MDA-MB-231 cells (two-tailed Student’s t test, *P<0.05, ***P<0.001). Each bar represented the mean ± SEM from three independent experiments. Scale bar=100 μm. Supplementary Fig. 3. Over-expression AQP1 increased breast cancer invasion abilities in T47D breast cancer cells. (a) Western blot analysis of the expr [file 13046_2023_2616_MOESM1_ESM.zip › Supplementary Fig. 3.tif]

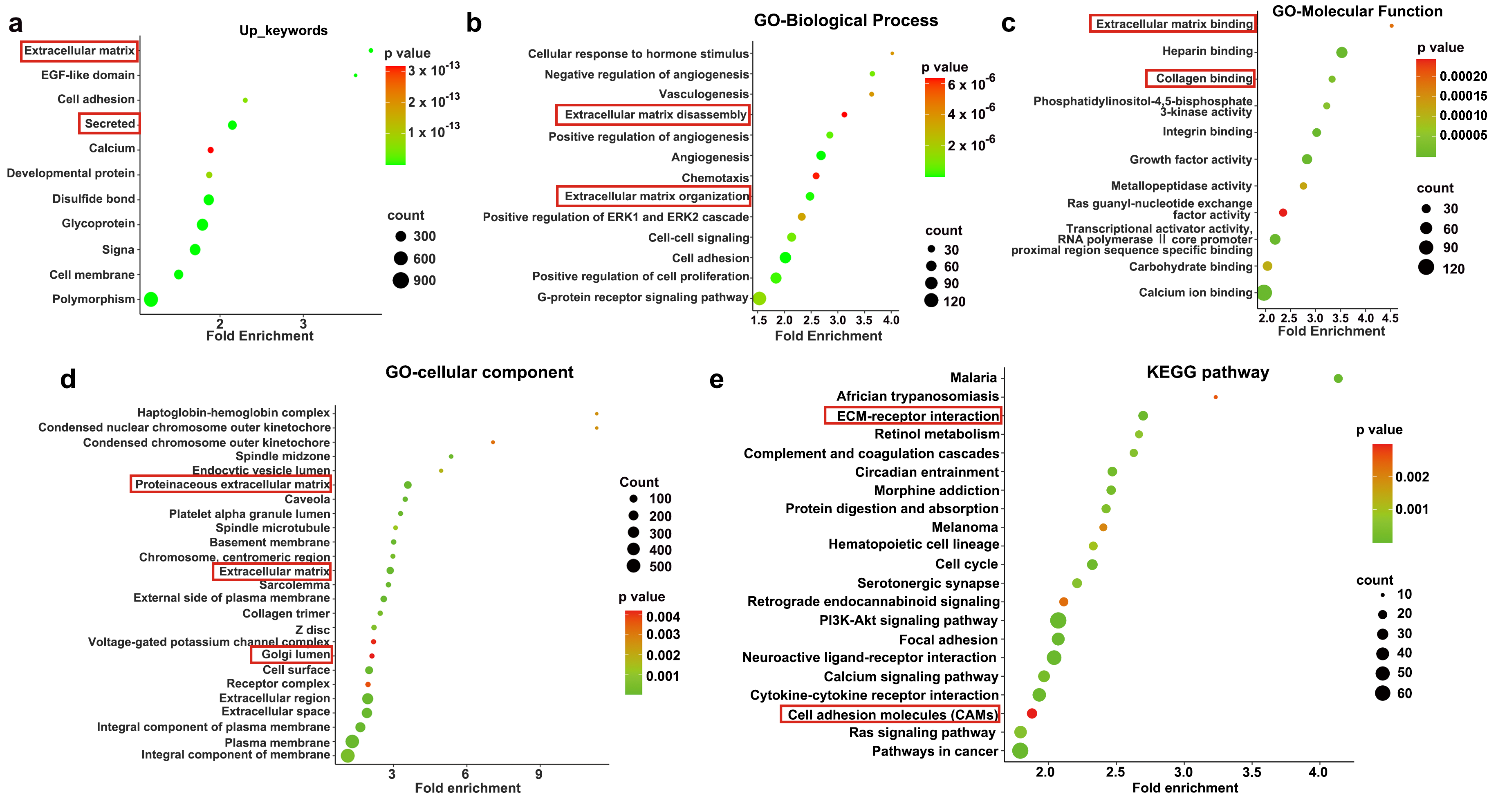

Supplement: Supplementary file 1 — Additional file 1: Supplementary Fig. 1. Cytoplasmic expression of AQP1 was positively correlated with breast cancer progression. (a) Patients who had a recurrence or metastasis had a higher AQP1 cytoplasmic expression (62.2% vs 38.9%, P = 0.009). Cyto-AQP1: cytoplasmic AQP1 expression. (b) Patients who had a lymph node metastasis (n > 4) had a higher AQP1 cytoplasmic expression (50.0% vs 38.1%, P = 0.038). Cyto-AQP1: cytoplasmic AQP1 expression. (c-d) The relationship between pT stage and AQP1 cytoplasmic expression. Cyto-AQP1: cytoplasmic AQP1 expression. (e) The tumor volume in Flag-vector/MDA-MB-231 and Flag-AQP1/MDA-MB-231 mice group. Values were expressed as mean ± SD (two-tailed Student’s t test and two-way ANOVA, **P < 0.01, ***P < 0.001). (f) Quantitation of the percentage of Ki67-positive cells in tumor sections of Flag-vector/MDA-MB-231 and Flag-AQP1/MDA-MB-231 mice group. Two-tailed Student’s t test, *P < 0.05. Supplementary Fig. 2. Down-regulated expression of AQP1 decreased breast cancer migration and invasion abilities in AQP1-overexpressing MDA-MB-231 cells. (a) Western blot analysis of the expression of AQP1 in Flag-AQP1/MDA-MB-231 cells transfected with AQP1 shRNA. GAPDH was the loading control. (b-c) The abilities of migration and invasion were detected using Flag-AQP1/MDA-MB-231 and Flag-AQP1/shAQP1/MDA-MB-231 cells. Values were expressed as mean ± SEM from three independent experiments (two-tailed Student’s t test, **P<0.01). Scale bar = 100 μm. (d-e) Migration and invasion assay showed that Flag-vector/MDA-MB-231 cells treated with the supernatant of Flag-AQP1/shAQP1/MDA-MB-231 cells reversed the promoted phenotype compared with Flag-AQP1/MDA-MB-231 cells (two-tailed Student’s t test, *P<0.05, ***P<0.001). Each bar represented the mean ± SEM from three independent experiments. Scale bar=100 μm. Supplementary Fig. 3. Over-expression AQP1 increased breast cancer invasion abilities in T47D breast cancer cells. (a) Western blot analysis of the expr [file 13046_2023_2616_MOESM1_ESM.zip › Supplementary Fig. 4.tif]

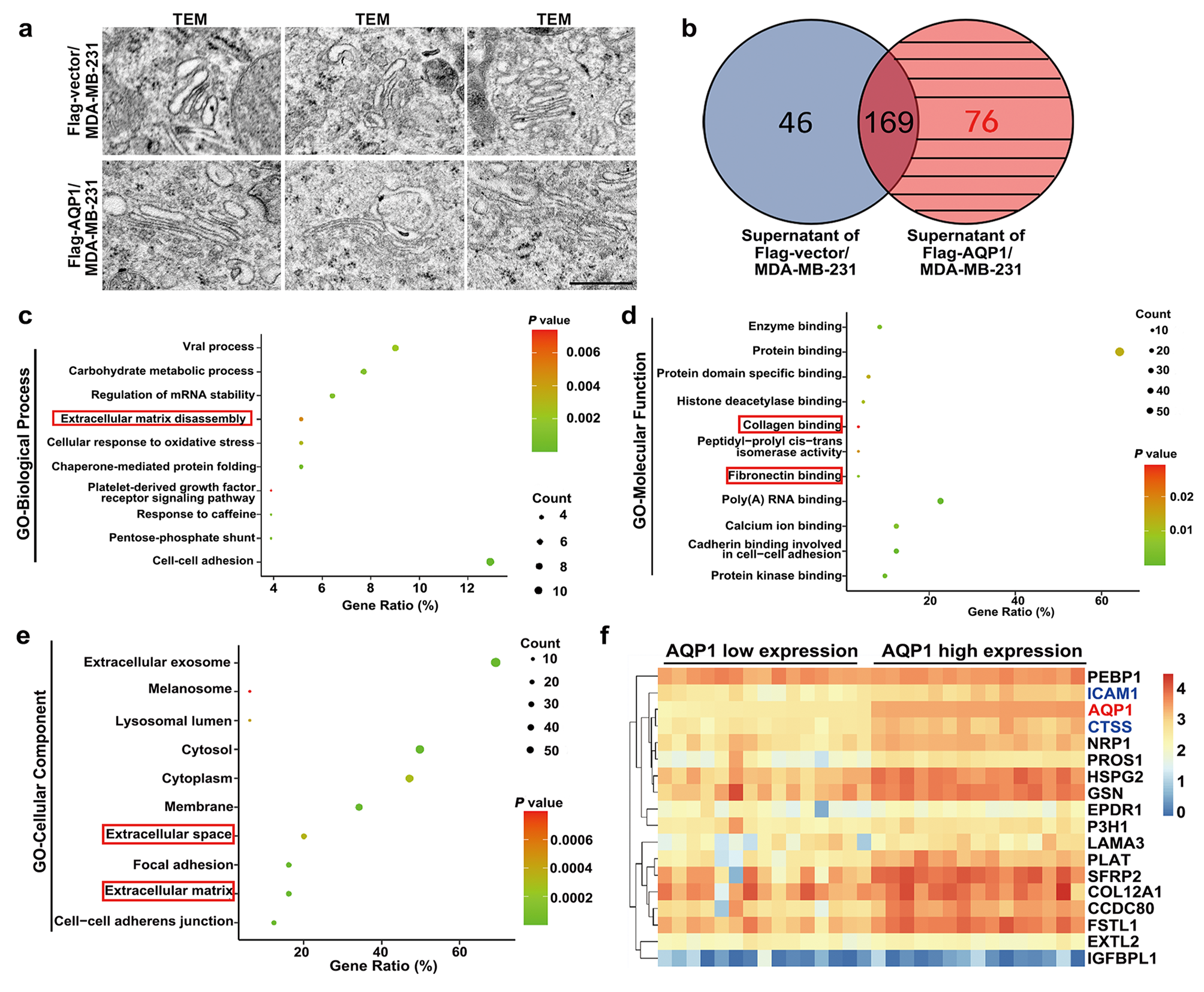

Supplement: Supplementary file 1 — Additional file 1: Supplementary Fig. 1. Cytoplasmic expression of AQP1 was positively correlated with breast cancer progression. (a) Patients who had a recurrence or metastasis had a higher AQP1 cytoplasmic expression (62.2% vs 38.9%, P = 0.009). Cyto-AQP1: cytoplasmic AQP1 expression. (b) Patients who had a lymph node metastasis (n > 4) had a higher AQP1 cytoplasmic expression (50.0% vs 38.1%, P = 0.038). Cyto-AQP1: cytoplasmic AQP1 expression. (c-d) The relationship between pT stage and AQP1 cytoplasmic expression. Cyto-AQP1: cytoplasmic AQP1 expression. (e) The tumor volume in Flag-vector/MDA-MB-231 and Flag-AQP1/MDA-MB-231 mice group. Values were expressed as mean ± SD (two-tailed Student’s t test and two-way ANOVA, **P < 0.01, ***P < 0.001). (f) Quantitation of the percentage of Ki67-positive cells in tumor sections of Flag-vector/MDA-MB-231 and Flag-AQP1/MDA-MB-231 mice group. Two-tailed Student’s t test, *P < 0.05. Supplementary Fig. 2. Down-regulated expression of AQP1 decreased breast cancer migration and invasion abilities in AQP1-overexpressing MDA-MB-231 cells. (a) Western blot analysis of the expression of AQP1 in Flag-AQP1/MDA-MB-231 cells transfected with AQP1 shRNA. GAPDH was the loading control. (b-c) The abilities of migration and invasion were detected using Flag-AQP1/MDA-MB-231 and Flag-AQP1/shAQP1/MDA-MB-231 cells. Values were expressed as mean ± SEM from three independent experiments (two-tailed Student’s t test, **P<0.01). Scale bar = 100 μm. (d-e) Migration and invasion assay showed that Flag-vector/MDA-MB-231 cells treated with the supernatant of Flag-AQP1/shAQP1/MDA-MB-231 cells reversed the promoted phenotype compared with Flag-AQP1/MDA-MB-231 cells (two-tailed Student’s t test, *P<0.05, ***P<0.001). Each bar represented the mean ± SEM from three independent experiments. Scale bar=100 μm. Supplementary Fig. 3. Over-expression AQP1 increased breast cancer invasion abilities in T47D breast cancer cells. (a) Western blot analysis of the expr [file 13046_2023_2616_MOESM1_ESM.zip › Supplementary Fig. 5.tif]

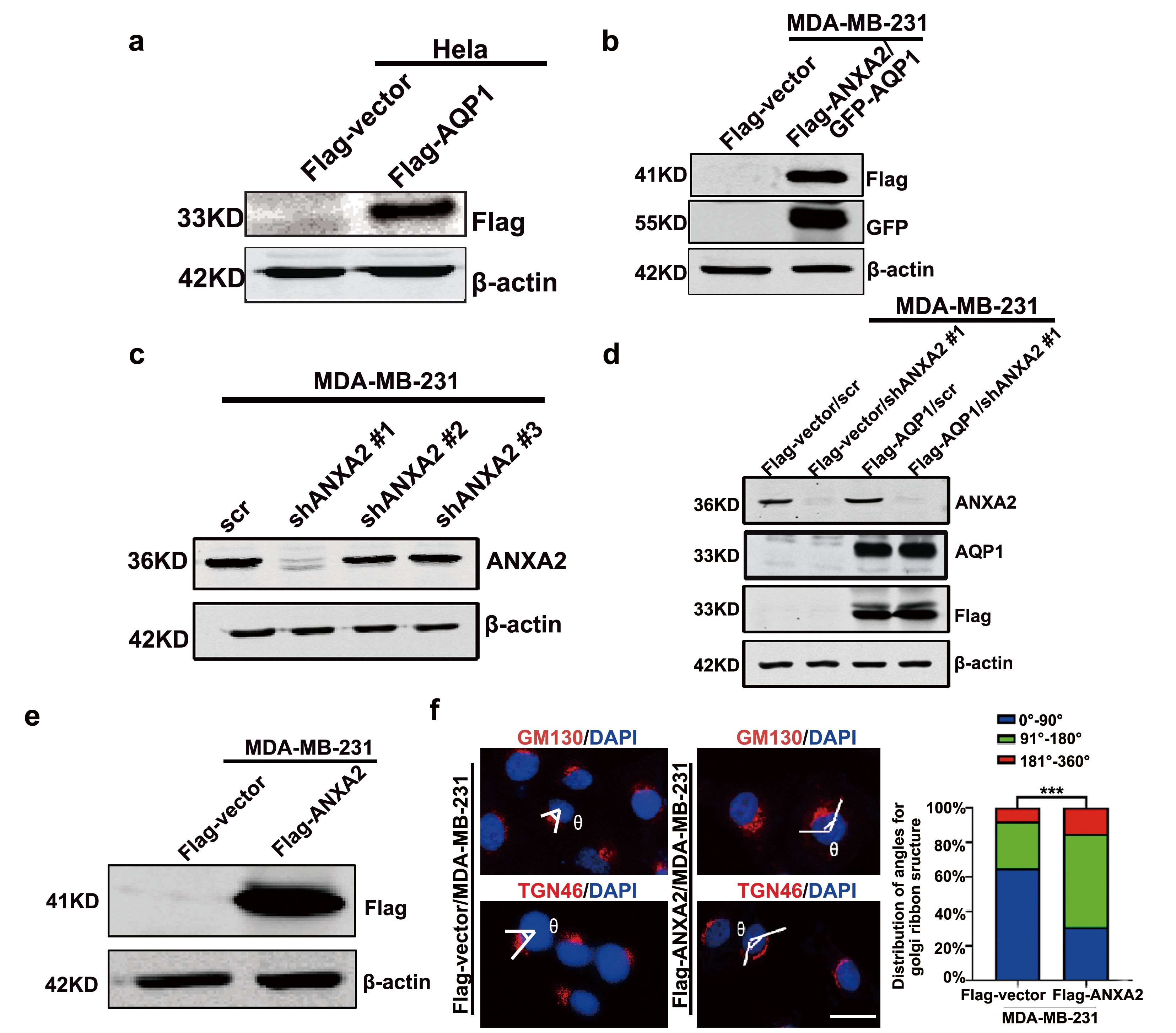

Supplement: Supplementary file 1 — Additional file 1: Supplementary Fig. 1. Cytoplasmic expression of AQP1 was positively correlated with breast cancer progression. (a) Patients who had a recurrence or metastasis had a higher AQP1 cytoplasmic expression (62.2% vs 38.9%, P = 0.009). Cyto-AQP1: cytoplasmic AQP1 expression. (b) Patients who had a lymph node metastasis (n > 4) had a higher AQP1 cytoplasmic expression (50.0% vs 38.1%, P = 0.038). Cyto-AQP1: cytoplasmic AQP1 expression. (c-d) The relationship between pT stage and AQP1 cytoplasmic expression. Cyto-AQP1: cytoplasmic AQP1 expression. (e) The tumor volume in Flag-vector/MDA-MB-231 and Flag-AQP1/MDA-MB-231 mice group. Values were expressed as mean ± SD (two-tailed Student’s t test and two-way ANOVA, **P < 0.01, ***P < 0.001). (f) Quantitation of the percentage of Ki67-positive cells in tumor sections of Flag-vector/MDA-MB-231 and Flag-AQP1/MDA-MB-231 mice group. Two-tailed Student’s t test, *P < 0.05. Supplementary Fig. 2. Down-regulated expression of AQP1 decreased breast cancer migration and invasion abilities in AQP1-overexpressing MDA-MB-231 cells. (a) Western blot analysis of the expression of AQP1 in Flag-AQP1/MDA-MB-231 cells transfected with AQP1 shRNA. GAPDH was the loading control. (b-c) The abilities of migration and invasion were detected using Flag-AQP1/MDA-MB-231 and Flag-AQP1/shAQP1/MDA-MB-231 cells. Values were expressed as mean ± SEM from three independent experiments (two-tailed Student’s t test, **P<0.01). Scale bar = 100 μm. (d-e) Migration and invasion assay showed that Flag-vector/MDA-MB-231 cells treated with the supernatant of Flag-AQP1/shAQP1/MDA-MB-231 cells reversed the promoted phenotype compared with Flag-AQP1/MDA-MB-231 cells (two-tailed Student’s t test, *P<0.05, ***P<0.001). Each bar represented the mean ± SEM from three independent experiments. Scale bar=100 μm. Supplementary Fig. 3. Over-expression AQP1 increased breast cancer invasion abilities in T47D breast cancer cells. (a) Western blot analysis of the expr [file 13046_2023_2616_MOESM1_ESM.zip › Supplementary Fig. 6.tif]

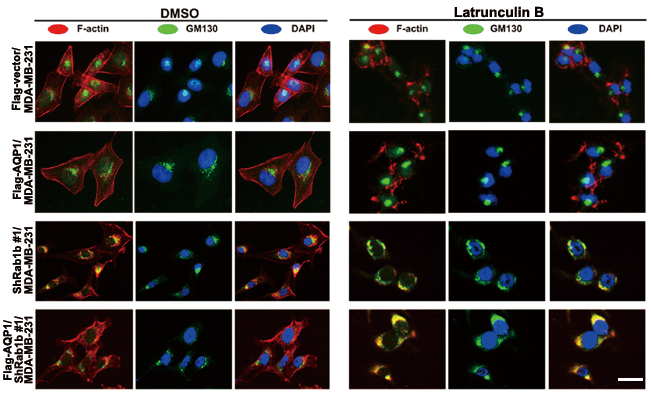

Supplement: Supplementary file 1 — Additional file 1: Supplementary Fig. 1. Cytoplasmic expression of AQP1 was positively correlated with breast cancer progression. (a) Patients who had a recurrence or metastasis had a higher AQP1 cytoplasmic expression (62.2% vs 38.9%, P = 0.009). Cyto-AQP1: cytoplasmic AQP1 expression. (b) Patients who had a lymph node metastasis (n > 4) had a higher AQP1 cytoplasmic expression (50.0% vs 38.1%, P = 0.038). Cyto-AQP1: cytoplasmic AQP1 expression. (c-d) The relationship between pT stage and AQP1 cytoplasmic expression. Cyto-AQP1: cytoplasmic AQP1 expression. (e) The tumor volume in Flag-vector/MDA-MB-231 and Flag-AQP1/MDA-MB-231 mice group. Values were expressed as mean ± SD (two-tailed Student’s t test and two-way ANOVA, **P < 0.01, ***P < 0.001). (f) Quantitation of the percentage of Ki67-positive cells in tumor sections of Flag-vector/MDA-MB-231 and Flag-AQP1/MDA-MB-231 mice group. Two-tailed Student’s t test, *P < 0.05. Supplementary Fig. 2. Down-regulated expression of AQP1 decreased breast cancer migration and invasion abilities in AQP1-overexpressing MDA-MB-231 cells. (a) Western blot analysis of the expression of AQP1 in Flag-AQP1/MDA-MB-231 cells transfected with AQP1 shRNA. GAPDH was the loading control. (b-c) The abilities of migration and invasion were detected using Flag-AQP1/MDA-MB-231 and Flag-AQP1/shAQP1/MDA-MB-231 cells. Values were expressed as mean ± SEM from three independent experiments (two-tailed Student’s t test, **P<0.01). Scale bar = 100 μm. (d-e) Migration and invasion assay showed that Flag-vector/MDA-MB-231 cells treated with the supernatant of Flag-AQP1/shAQP1/MDA-MB-231 cells reversed the promoted phenotype compared with Flag-AQP1/MDA-MB-231 cells (two-tailed Student’s t test, *P<0.05, ***P<0.001). Each bar represented the mean ± SEM from three independent experiments. Scale bar=100 μm. Supplementary Fig. 3. Over-expression AQP1 increased breast cancer invasion abilities in T47D breast cancer cells. (a) Western blot analysis of the expr [file 13046_2023_2616_MOESM1_ESM.zip › Supplementary Fig. 7.tif]

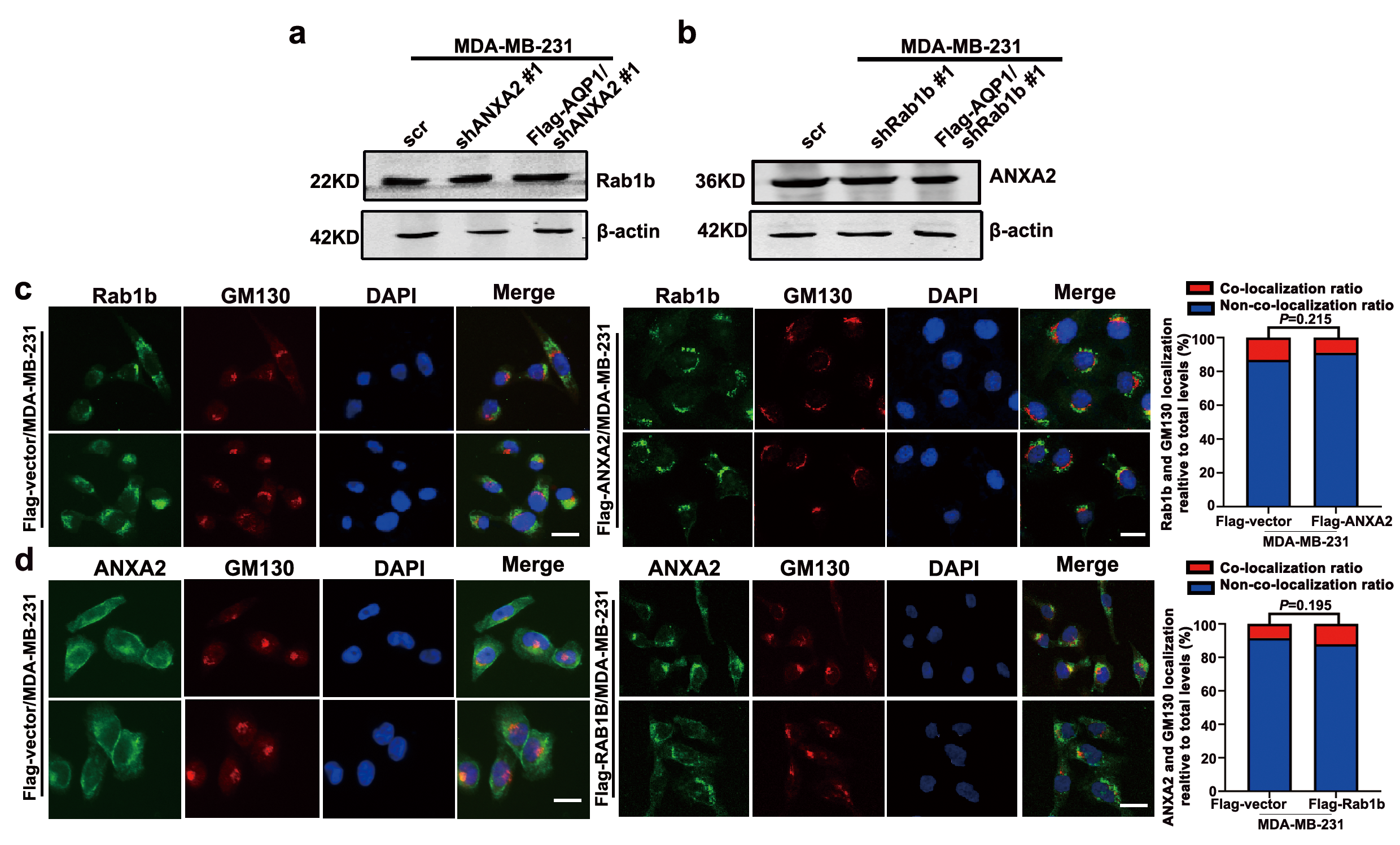

Supplement: Supplementary file 1 — Additional file 1: Supplementary Fig. 1. Cytoplasmic expression of AQP1 was positively correlated with breast cancer progression. (a) Patients who had a recurrence or metastasis had a higher AQP1 cytoplasmic expression (62.2% vs 38.9%, P = 0.009). Cyto-AQP1: cytoplasmic AQP1 expression. (b) Patients who had a lymph node metastasis (n > 4) had a higher AQP1 cytoplasmic expression (50.0% vs 38.1%, P = 0.038). Cyto-AQP1: cytoplasmic AQP1 expression. (c-d) The relationship between pT stage and AQP1 cytoplasmic expression. Cyto-AQP1: cytoplasmic AQP1 expression. (e) The tumor volume in Flag-vector/MDA-MB-231 and Flag-AQP1/MDA-MB-231 mice group. Values were expressed as mean ± SD (two-tailed Student’s t test and two-way ANOVA, **P < 0.01, ***P < 0.001). (f) Quantitation of the percentage of Ki67-positive cells in tumor sections of Flag-vector/MDA-MB-231 and Flag-AQP1/MDA-MB-231 mice group. Two-tailed Student’s t test, *P < 0.05. Supplementary Fig. 2. Down-regulated expression of AQP1 decreased breast cancer migration and invasion abilities in AQP1-overexpressing MDA-MB-231 cells. (a) Western blot analysis of the expression of AQP1 in Flag-AQP1/MDA-MB-231 cells transfected with AQP1 shRNA. GAPDH was the loading control. (b-c) The abilities of migration and invasion were detected using Flag-AQP1/MDA-MB-231 and Flag-AQP1/shAQP1/MDA-MB-231 cells. Values were expressed as mean ± SEM from three independent experiments (two-tailed Student’s t test, **P<0.01). Scale bar = 100 μm. (d-e) Migration and invasion assay showed that Flag-vector/MDA-MB-231 cells treated with the supernatant of Flag-AQP1/shAQP1/MDA-MB-231 cells reversed the promoted phenotype compared with Flag-AQP1/MDA-MB-231 cells (two-tailed Student’s t test, *P<0.05, ***P<0.001). Each bar represented the mean ± SEM from three independent experiments. Scale bar=100 μm. Supplementary Fig. 3. Over-expression AQP1 increased breast cancer invasion abilities in T47D breast cancer cells. (a) Western blot analysis of the expr [file 13046_2023_2616_MOESM1_ESM.zip › Supplementary Fig. 8.tif]

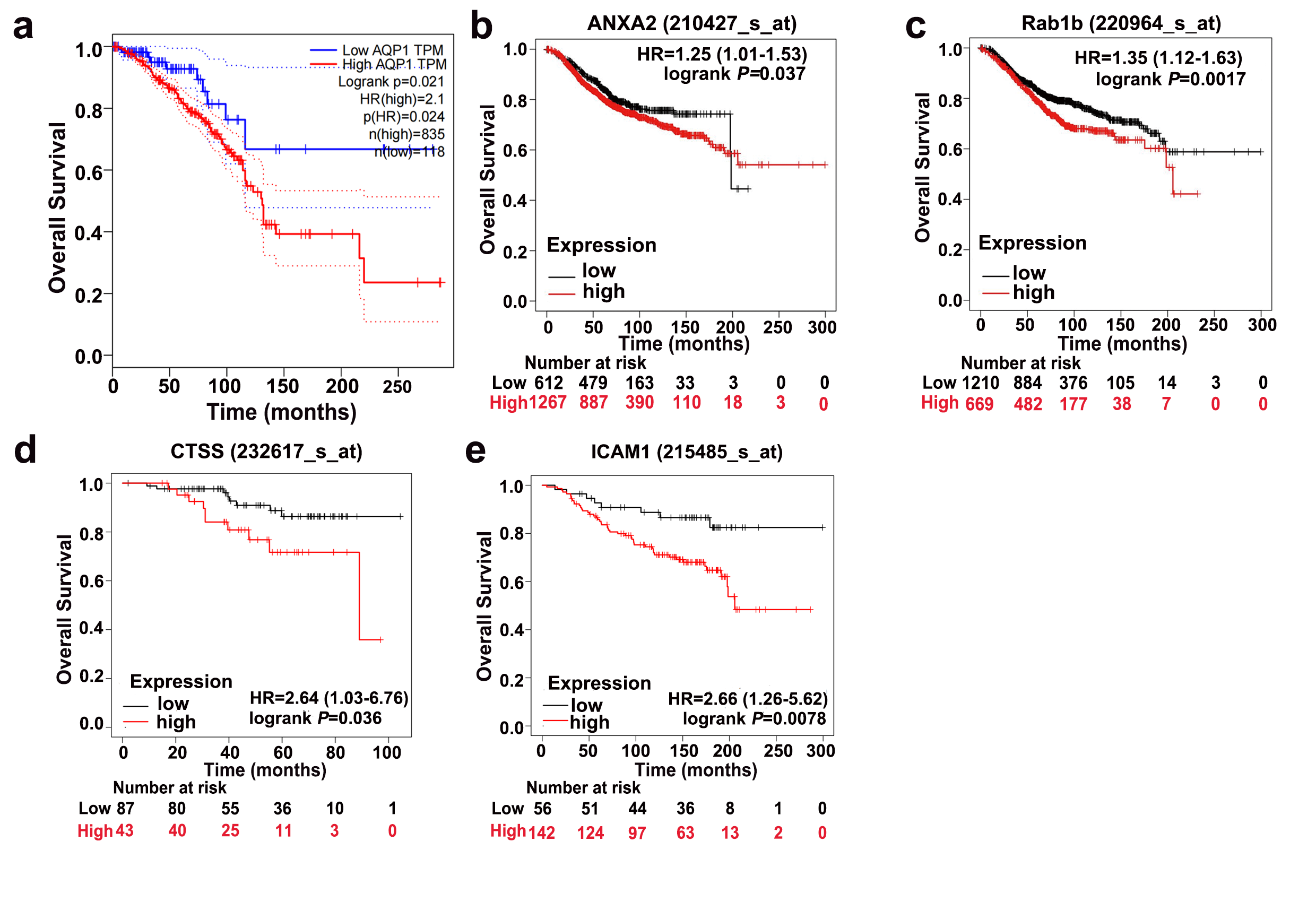

Supplement: Supplementary file 1 — Additional file 1: Supplementary Fig. 1. Cytoplasmic expression of AQP1 was positively correlated with breast cancer progression. (a) Patients who had a recurrence or metastasis had a higher AQP1 cytoplasmic expression (62.2% vs 38.9%, P = 0.009). Cyto-AQP1: cytoplasmic AQP1 expression. (b) Patients who had a lymph node metastasis (n > 4) had a higher AQP1 cytoplasmic expression (50.0% vs 38.1%, P = 0.038). Cyto-AQP1: cytoplasmic AQP1 expression. (c-d) The relationship between pT stage and AQP1 cytoplasmic expression. Cyto-AQP1: cytoplasmic AQP1 expression. (e) The tumor volume in Flag-vector/MDA-MB-231 and Flag-AQP1/MDA-MB-231 mice group. Values were expressed as mean ± SD (two-tailed Student’s t test and two-way ANOVA, **P < 0.01, ***P < 0.001). (f) Quantitation of the percentage of Ki67-positive cells in tumor sections of Flag-vector/MDA-MB-231 and Flag-AQP1/MDA-MB-231 mice group. Two-tailed Student’s t test, *P < 0.05. Supplementary Fig. 2. Down-regulated expression of AQP1 decreased breast cancer migration and invasion abilities in AQP1-overexpressing MDA-MB-231 cells. (a) Western blot analysis of the expression of AQP1 in Flag-AQP1/MDA-MB-231 cells transfected with AQP1 shRNA. GAPDH was the loading control. (b-c) The abilities of migration and invasion were detected using Flag-AQP1/MDA-MB-231 and Flag-AQP1/shAQP1/MDA-MB-231 cells. Values were expressed as mean ± SEM from three independent experiments (two-tailed Student’s t test, **P<0.01). Scale bar = 100 μm. (d-e) Migration and invasion assay showed that Flag-vector/MDA-MB-231 cells treated with the supernatant of Flag-AQP1/shAQP1/MDA-MB-231 cells reversed the promoted phenotype compared with Flag-AQP1/MDA-MB-231 cells (two-tailed Student’s t test, *P<0.05, ***P<0.001). Each bar represented the mean ± SEM from three independent experiments. Scale bar=100 μm. Supplementary Fig. 3. Over-expression AQP1 increased breast cancer invasion abilities in T47D breast cancer cells. (a) Western blot analysis of the expr [file 13046_2023_2616_MOESM1_ESM.zip › Supplementary Fig. 9.tif]
