# Supplementary material for: Water channel protein AQP1 in cytoplasm is a critical factor in breast cancer local invasion
Source: J Exp Clin Cancer Res. 2023 Feb 20;42:49. doi: 10.1186/s13046-023-02616-1 (PMC9940370; doi:10.1186/s13046-023-02616-1)
Supplement: Supplementary file 2 — Additional file 2: Supplementary Table S1. List of plasmids and RNA interference sequences used in this study. Supplementary Table S2. List of antibodies used in this study. Supplementary Table S3. Cytoplasmic AQP1 expression in IDC patients. Supplementary Table S4. Relationship between AQP1 cytoplasmic expression and pathological tumor size (pT). Supplementary Table S5. Univariate and multivariate analysis for overall survival (OS) and progression-free survival (PFS). Supplementary Table S6. List of proteins in the supernant of Flag-vector/MDA-MB-231 cells by mass spectrometry. Supplementary Table S7. List of proteins in the supernant of Flag-AQP1/MDA-MB-231 cells by mass spectrometry. Supplementary Table S8. Correlations among the expression of AQP1, ANXA2 and CTSS in 194 IDC patients. Supplementary Table S9. Relationship between AQP1 cytoplasmic expression and ANXA2 membrane expression in IDC patients (n=194). Supplementary Table S10. List of 23 genes of Rab family associated with AQP1 and ANXA2 by analysis of TCGA database. [file 13046_2023_2616_MOESM2_ESM.zip › Supplementary Table S8.docx]

**Supplementary Table S8. Correlations among the expression of AQP1, ANXA2 and CTSS in 194 IDC patients.**

| **Variable**  **(rate of high expression)** | **AQP1**  **(110/194, 56.7%)** | **Cytoplasmic ANXA2**  **(96/194, 49.5%)** | **CTSS**  **(86/194, 44.3%)** |
| --- | --- | --- | --- |
| **AQP1** |  | ***r*_s_ =0.363**  ***P*= 0.000** | ***r*_s_ =0.204**  ***P*= 0.004** |

***P* value was calculated by Spearman’s Rank-Correlation test.**
